# Diversity and Oil Degradation Potential of Culturable Microbes Isolated from Chronically Contaminated Soils in Trinidad

**DOI:** 10.3390/microorganisms9061167

**Published:** 2021-05-28

**Authors:** Amanda C. Ramdass, Sephra N. Rampersad

**Affiliations:** Biochemistry Research Laboratory (Rm216), Department of Life Sciences, Faculty of Science and Technology, The University of the West Indies, Trinidad and Tobago, West Indies; ac_ramdass@hotmail.com

**Keywords:** bioremediation, species diversity, lipase, oil-degrading microorganisms

## Abstract

Trinidad and Tobago is the largest producer of oil and natural gas in Central America and the Caribbean. Natural crude oil seeps, in addition to leaking petroleum pipelines, have resulted in chronic contamination of the surrounding terrestrial environments since the time of petroleum discovery, production, and refinement in Trinidad. In this study, we isolated microbes from soils chronically contaminated with crude oil using a culture-dependent approach with enrichment. The sampling of eight such sites located in the southern peninsula of Trinidad revealed a diverse microbial composition and novel oil-degrading filamentous fungi and yeast as single-isolate degraders and naturally occurring consortia, with specific bacterial species not previously reported in the literature. Multiple sequence comparisons and phylogenetic analyses confirmed the identity of the top degraders. The filamentous fungal community based on culturable species was dominated by Ascomycota, and the recovered yeast isolates were affiliated with Basidiomycota (65.23%) and Ascomycota (34.78%) phyla. Enhanced biodegradation of petroleum hydrocarbons is maintained by biocatalysts such as lipases. Five out of seven species demonstrated extracellular lipase activity in vitro. Our findings could provide new insights into microbial resources from chronically contaminated terrestrial environments, and this information will be beneficial to the bioremediation of petroleum contamination and other industrial applications.

## 1. Introduction

Trinidad and Tobago is the largest producer of oil and natural gas in the Caribbean and Central America [1,2]. As a result of its large reserves of oil and natural gas, exploitation of the latter now drives its economy. As such, Trinidad has one of the largest natural gas processing facilities in the Western Hemisphere. Trinidad was subject to more than a century of petroleum exploration, which began in 1857 and was based on the detection of oil discharge in terrestrial environments identified as natural crude oil seeps in the southwestern peninsula of the island [3]. The largest naturally-occurring petroleum seep in the world is the Pitch Lake in Trinidad, which was described in 1595 by Sir Walter Raleigh in his search for El Dorado. Current seepage detection by SAR (synthetic aperture radar) also indicates multiple seepage sites offshore [4]. Natural crude oil seeps, in addition to leaking petroleum pipelines, have resulted in the contamination of the surrounding terrestrial environments since the time of petroleum discovery, production, and refinement. It is hypothesized that the chronically contaminated sites in Trinidad are inhabited by novel, indigenous microorganisms with generalist, as well as specialist, metabolic functions for utilizing crude oil as a carbon source, which are required for sustaining the microbial community in this type of perturbed terrestrial ecosystem. Sutton et al. [5] indicated that conclusions based on the short-term simulated spiking of soils may not reflect long-term natural remediation conditions in situ; the authors support the need to investigate long-term contaminated sites.

Crude oil is a heterogeneous mixture composed of saturates, aromatic hydrocarbons (including polycyclic aromatic hydrocarbons, PAHs), asphaltenes, and resins [6,7] that are hazardous to animal and human health [8,9,10]. Of particular concern is the percolation of surface spills on land (leachates) and the subsequent partitioning into gaseous, aqueous, and pure phases in the subsurface strata and migration of BTEX (benzene, ethylbenzene, toluene, and xylene) components of crude oil into waterways, aquifers, and water tables, which result in subsurface persistence and pose long-term effects on groundwater quality [11,12,13,14,15,16]. PAHs are chemically stable, demonstrate extreme persistence in contaminated environments, and are proven to be carcinogenic, genotoxic, teratogenic, and mutagenic [17,18,19]. Remediation of oil-contaminated terrestrial environments is focused on physio-chemical and/or biological methods for restoration [20]. Bioremediation methods exploit the use of microorganisms and their diverse metabolic capabilities for the attenuation of the toxic effects of hydrocarbon chemicals by various routes, including transforming them into less toxic products than the parent compound, completely mineralizing and/or immobilizing them [10,21,22,23]. Bioremediation is considered to be ‘environmentally benign’ or ‘passive’ and economically feasible compared to other approaches that are more costly and offer lower levels of efficacy [12].

Studies on communities of hydrocarbon-utilizing microorganisms have indicated that, over time, degradation will occur without external intervention via a process of ‘natural attenuation’ [5,24]. In simulated spiking experiments, the addition of different amounts of oil to soil resulted in selection for specific oil-degrading fungal populations with a concomitant reduction of species diversity and, in some cases, the complete elimination of other species [25]. This reduction in species diversity with increasing concentrations of supplemented crude oil may be explained by sublethal effects on these microbes through the bioconcentration of toxic pollutants and the creation of anoxic conditions of growth. It has also been documented that yeast and filamentous fungi sequester hydrocarbons in the cytoplasm of their cells [26].

Wang et al. [27] concluded that consequent to PAH contamination, there is a commonly occurring pattern of reduction in the number of and species diversity of bacteria as a result of selection pressures unique to oil-contaminated sites. In fact, such changes in selective pressure to favor the enrichment of those microbial species that are physiologically and metabolically adapted to hydrocarbon utilization often result in population shifts in the dominant species, which can, in turn, define the community‘s overall structure at any given point in time. Other studies have provided evidence that subsurface geochemical properties also influence biodegrading populations of microorganisms because these characteristics affect the bioavailability of PAHs as carbon sources to these microorganisms [28,29].

The relative contribution of culturable microbes to remediation is still studied using pure cultures, even though a large proportion of microbes remain unculturable [30,31]. In contrast, the culture-independent approach focuses on identifying genes associated with metabolic function [32,33,34] but does little to describe or provide proof of function of such microbes in situ. Furthermore, studies have indicated that the identified microbial composition and diversity may vary according to the mode of identification and characterization, i.e., culture-dependent vs. culture-independent methods [35]. This difference is also important in chronically contaminated soil, which, after decades of in situ enrichment, maintains a diverse range of oil-degrading microorganisms (some with specialized functions) that provides a unique collection of species for isolating crude oil-degrading microorganisms [36,37,38,39]. Wang, Li, Zhan, and Zhu [27] added that cooperative interactions among such microbial species are necessary in order to efficiently utilize crude oil and polycyclic aromatic hydrocarbons as a carbon source in contaminated soil [40,41].

Fungi are particularly good candidates for remediation because (i) they have long-range transportation systems—the movement of hyphae is not limited by a hydrophobic environment, surpassing air–water interfaces and air-filled pores in soil, where they aid in the transport of extrahyphal bacteria that would otherwise be limited by this physical barrier, (ii) fungal hyphae can function in anoxic conditions, which is often the case with thick oil slicks, (iii) they can tolerate extreme environments—numerous fungi are xero- and osmotolerant and can survive in a pH range of 1 to 9 and at temperatures of 5 to 60 °C, (iv) numerous fungi have extracellular enzymes that can metabolize hydrocarbons at the start of degradation, thus aiding the process, and (v) they are capable of catabolizing recalcitrant hydrocarbons, i.e., they can metabolize and mineralize high molecular weight hydrocarbons such as PAHs, unlike bacteria (because of the low bioavailability of these compounds to bacteria) [42,43,44,45]. Extracellular enzymatic transformation of recalcitrant compounds by fungi, followed by bacterial degradation of the subsequent intermediates produced through fungal action, may contribute to a combinatorial strategy for the biodegradation of PAHs [46].

As a result of a decline in microbial diversity, culture-dependent sampling of hydrocarbon-contaminated soils compared to other types of polluted sites may be more suitable than other approaches because a reduced sampling effort may still reflect a representative proportion of the bioactive microbial community [47,48,49]. Hydrocarbon contaminants will suppress the survival of PAH-sensitive bacterial groups [50] and select primarily for key subgroups of PAH-degrading bacteria (e.g., α- and γ-proteobacteria) in affected soils [51,52]. Culture-dependent methods are still a critical component of bioremediation development and research even though they generally recover a small portion of the diversity from soil environments compared to metagenomics analyses [53]. Among the advantages of culture-dependent methods are the potential use of cultured microbes and their products in in situ and ex situ applications. Additionally, microbial isolation enables in vitro assessments of isolate hydrocarbon degradation pathways and oil-degrading capability, thereby providing a basis for identifying genes that could be useful in land remediation [35]. Using traditional selective cultivation-dependent microbiological methods also allows the isolation of site-specific hydrocarbonoclastic bacteria [35]. Metagenomics may not be able to detect rarer bacteria or the DNA of both dead and living cells and, ultimately, does not provide any practical means of recovering live microbes. Given the century-old history of crude oil production in Trinidad, it is hypothesized that unique indigenous microorganisms can survive and out-compete other microbes in using crude oil as a carbon source in oil-contaminated soils.

In view of the current evidence, it is, therefore, important to assess the co-aggregation or co-colonization and niche overlap in chronically contaminated terrestrial ecosystems inhabited by microorganisms. Empirical evidence indicates that bioremediation treatments should be designed to be site-specific and fundamentally guided by an assessment of the localized and culturable microbial populations in terms of their biodiversity, relative abundance, and species richness. Conjunctive to this approach is the challenge to cultivate these interacting microorganisms and characterize their function in vitro.

Therefore, the main objectives of this study are: (i) to isolate culturable filamentous fungi and yeast from eight naturally occurring, hydrocarbon-contaminated sites in southern Trinidad; (ii) to assess the ability of indigenous isolates to utilize crude oil as a unique carbon source in vitro; (iii) to identify these PAH-degrading isolates using molecular techniques; (iv) to determine the ability of selected isolates to produce extracellular lipases and; (v) to evaluate the relative microbial composition, species diversity, and possible community ordination at each site. Whereas other studies have examined microbial populations in artificially spiked soils to simulate contaminated microcosms, this study examines field-collected samples collected at geographically distinct sites with different soil matrices and contamination levels. This is the first extensive study to isolate microbes with the potential for bioremediation and the ability to produce bioactive compounds from sites not previously explored in south Trinidad.

## 2. Materials and Methods

### 2.1. Site Descriptions

**Figure 1:**
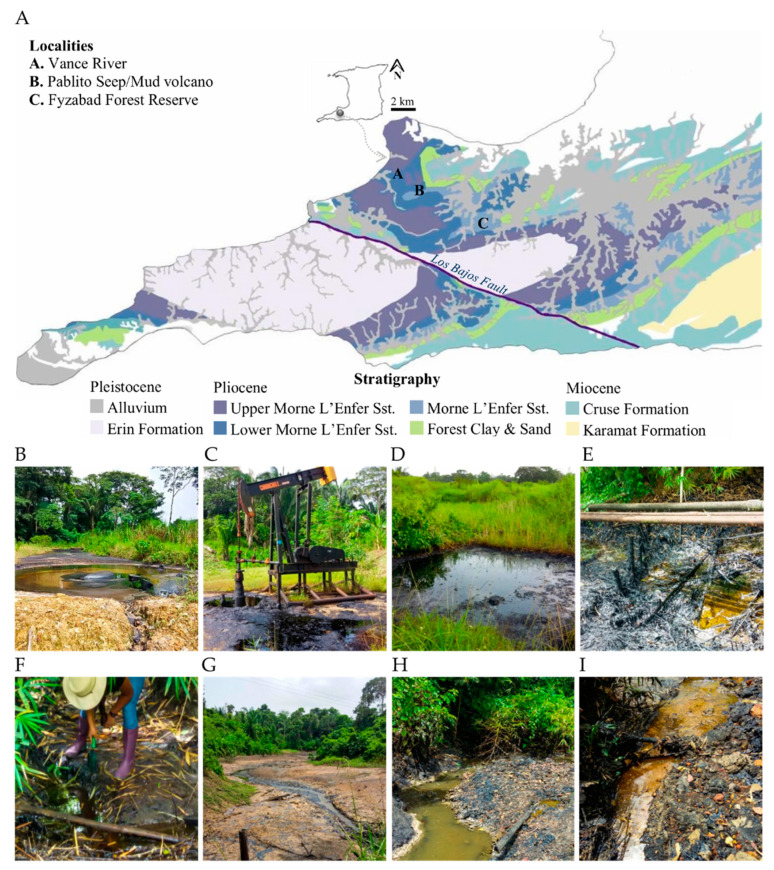
Sampling site description. (**A**): Locations of the sampling sites in southwestern Trinidad. Sites were concentrated in Plio-Pleistocene formations, with surrounding active oil fields and seeps. Letters on the map represent the localities mentioned in the text, and the colors represent various geological formations. Geological formations are redrawn from Kugler [54], Chen et al. [55], Dasgupta et al. [56], and Baboolal et al. [57]. The 8 sampling sites in the 3 geographically separated locations in southwestern Trinidad: (**B**): Pablito P1; (**C**): Fyzabad F1; (**D**): Fyzabad F2; (**E**): Vance river V1; (**F**): Vance river V2; (**G**): Vance river V3; (**H**): Vance river V4; (**I**): Vance river V5. Photographs by AC Ramdass and SN Rampersad.

Eight study sites in three geographically separated localities in southern Trinidad were selected (Figure 1 and Table 1). These sites are associated with oil accumulations in the Miocene and Pliocene reservoirs, where crude oil has a historical occurrence. The first location, Pablito (P), was selected within the grassland area of a tropical evergreen forest (10° 11′ 37.2516″ N, 61° 37′ 13.8972″ W). One site in P containing a natural oil seep/mud volcano was sampled (Figure 1B). The second location, Fyzabad (F), is located in a tropical evergreen forest, the Fyzabad Forest Reserve (10° 10′ 0.01″ N, 61° 30′ 0″ W). Two geographically isolated sites in F, an abandoned well and a natural oil seep, were selected for sampling as an anthropogenic feature (Figure 1C,D). The third location was Vance River, Vance (V), (10° 12′ 06.2″ N, 61° 37′52.2″ W). Five geographically isolated sites were sampled at Vance River, including oil-contaminated sediments surrounding leaking pipelines, a natural oil seep where the surrounding land was heavily impregnated with oil, and sites along the river containing oil runoff accumulated in sediments (Figure 1E–I).

In Trinidad, significant asphaltic deposits are found in the southwestern region of the island, concentrated in Pilo-Pleistocene formations. For this study, we choose three unique asphaltic localities within these reservoirs. Pablito, Fyzabad, and Vance share their reservoirs with the same hydrocarbon source, the Pliocene-aged Morne L’Enfer formation sandstone and silt [2,59]. At the surface of this formation, several heavy oil and oil sand occurrences are common, and their exposure allowed for the opportunity to study the microbial communities present in heavy oil-contaminated sediments.

One site containing a natural seep is located in Pablito. This semi-evergreen seasonal forest locality is known to contain both asphalt seeps/mud volcanoes. There have been high levels of seep activity. There is no information, to our knowledge, about the physical environment in terms of soil composition, however, because it is a tropical forest, the soil pH would be slightly acidic to neutral [60]. The Fyzabad Forest Reserve is characterized as having a dense tropical semi-evergreen seasonal forest that has had minimal human disturbance since the private oilfield road spanning the reserve was gated off from the general public. The soil is the typical pH of tropical forest, slightly acidic to neutral [60]. Vance River lacks anthropogenic contamination since it is remote from settled areas.

Reservoir properties for these sites have been previously investigated [58] and are outlined in Table 1. The features and formation of these sites suggest that reservoirs are ideal for this study.

### 2.2. Soil Sampling and Isolation of Culturable Microbes

Samples of mud and oil within 0.5 m to crude oil sources were collected in September 2017. Soil and oil samples were collected at a depth of 0 to 10 cm using a stainless steel shovel and placed into Sterile Whirl-Pak bags that were sealed immediately after collection to avoid contamination. Soil samples were transported to the laboratory at 4 °C following collection, and microbial isolation was done within 24 h.

Microbes were isolated by means of serial dilution technique. For each soil sample, 1 g of soil was suspended in 9 mL sterile phosphate buffer pH 7.2 (KH_2_PO_4_ 68 g/L) (Sigma-Aldrich, St. Louis, MO, USA), and a 100 μL aliquot from dilutions 10^−1^ to 10^−3^ were spread on a petri dish containing potato dextrose agar (PDA, HiMedia Laboratories LLC., West Chester, PA, USA) supplemented with 50 mg/L each of streptomycin and tetracycline (Sigma-Aldrich, St. Louis, MO, USA) to inhibit bacterial growth. All Petri dishes were incubated at 25 °C in the dark for a period of five days. Fungal, yeast, and antibiotic-resistant bacteria were then isolated in pure culture for further use. Inverted cultures were maintained on different media and incubated (27 °C for bacteria and 25 °C for fungi). Bacterial colonies were checked every 48 h for six days, and each morphotype was subcultured on PDA and Reasoner’s 2A agar (R2A, HiMedia Laboratories LLC., West Chester, PA, USA) for 48 h. R2A is designed to promote the growth of microorganisms that grow in low nutrient environments (Sigma-Aldrich, St. Louis, MO, USA). Fungal colonies were checked every day for two weeks and were subcultured on PDA for three weeks. Bacterial and fungal pure cultures were stored at 4 °C until gDNA extraction. In some cases, the isolates used in this study existed as co-cultures since successive subculturing to obtain pure isolates failed, and fungal–bacterial and yeast–bacterial co-cultures were included in the study.

### 2.3. Isolation of Crude Oil-Degrading Microbes

All isolated fungi, bacteria, and yeast were screened for their ability to utilize crude oil as a carbon source in vitro. Candidate crude oil metabolizing microbes were isolated using a growth bioassay on crude-oil-amended plates. Assays were prepared using Bushnell-Haas agar (BHA, Sigma-Aldrich, St. Louis, MO, USA) to provide the proper microelement amounts for microbial growth, except a carbon source. BHA is recommended for the examination of fuels for microbial contamination and for studying hydrocarbon deterioration by microorganisms (Sigma-Aldrich, St. Louis, MO, USA). BHA media was supplemented with crude oil (2% *v*/*v*; CARIRI Laboratories, St. Augustine, Trinidad and Tobago) and 50 mg/L each of streptomycin and tetracycline. Duplicate assays were prepared under aseptic conditions. In order to obtain homogenous distribution of oil, the media was thoroughly mixed by hand before it was added to each plate. All plates were inoculated with a 4 mm^3^ block of mycelium taken from the advancing edge of a colony of an actively growing axenic culture. The isolates showing growth on these assays were then characterized after two to five days of incubation at 25 °C in the dark.

### 2.4. Characterization of Growth on Crude Oil In vitro

Isolates showing growth on crude oil assays were characterized for their ability to grow on crude oil. Fungi and yeast (existing as co-cultures) were characterized in vitro on BHA (as described above), and, for luxuriant growth, isolates were also screened on PDA. Bacterial isolates (existing as co-cultures or pure isolates) were screened using R2A media. The media used was supplemented with crude oil (2% *v*/*v*), 50 mg/L each of streptomycin and tetracycline for fungal and yeast assays, and with and without these antibiotics for the bacterial assays.

To characterize the crude-oil-utilizing microbes, growth assays were conducted by comparing the growth rates of colonies after 6 days in addition to the appearance of the colony on the plates for fungi and yeast, i.e., (i) the zone of clearance of oil around the colony, and (ii) the disappearance of oil seen on the reverse view of the colony. The top performers were considered the most efficient hydrocarbon-utilizing microbes, and these were selected for identification and further study.

### 2.5. Separation of Yeast–Bacteria Co-Cultures

Isolates F1 6, F1 7, and F1 9, existing in co-cultures, were separated to obtain pure cultures to perform lipase assays. Isolation of the pure bacteria *Chryseobacterium oranimense* (F1 6), existing in bacteria–yeast co-culture, was achieved by successive subculturing by streaking the isolate onto specialized media consisting of MacConkey agar (HiMedia Laboratories LLC., West Chester, PA, USA) containing 0.1 mg/L cycloheximide (Sigma-Aldrich, St. Louis, MO, USA) and adjusted to pH 8.5 with 1 M NaOH (Sigma-Aldrich, St. Louis, MO, USA). After several weeks, the pure bacterial colony was plated on MacConkey plates with no fungicide, and DNA was extracted, amplified, sequenced, and identified, as described below, to confirm the presence of the bacteria alone. Isolation of *Rhodotorula mucilaginosa* (F1 7) and *Lecythophora aff. decumbens* (F1 9) yeast as pure cultures from the bacterial–yeast co-cultures was achieved by successive subculturing of isolates onto specialized media that was designed using key factors that inhibit bacterial growth, including low pH, high salinity, the use of multiple antibiotics, and a high concentration of glucose, to aid separation since initial attempts with common methods failed. Modified yeast malt agar (YM, HiMedia Laboratories LLC., West Chester, PA, USA) and glucose (Sigma-Aldrich, St. Louis, MO, USA) were prepared. Briefly, NaCl (7% *w*/*v*) was dissolved in double-distilled water; YM was then added, and the solution was sterilized. Once cooled, the pH was adjusted to approximately 3.5 using 1 N HCl (Sigma-Aldrich, St. Louis, MO, USA), and 50 mg/L each of streptomycin and tetracycline was added to the media. Plates were streaked with a co-culture colony, and, after successive subculturing, the pure yeast colony was plated on modified YM agar, and DNA was extracted, amplified, sequenced, and identified, as described below, to ensure the culture was indeed pure.

### 2.6. Selection of Efficient Hydrocarbon-Utilizing Microbes

Isolates showing maximum potential and top performance in vitro in crude oil assays were selected for molecular identification. The criteria for selection included: (i) minimum diameter of growth on BHA of at least 2/3 of the growth on PDA for fungal isolates, (ii) isolates must show a level of oil-degrading ability, i.e., significant zone of clearance, disappearance of crude oil, and (iii) novel species not previously reported in the literature. Isolates were maintained on appropriate agar plates (for fungi and yeast: PDA and BHA; for bacterial co-cultures: PDA and R2A) and in 1.5 mL centrifuge tubes (Eppendorf, Hamburg, Germany) in sterile distilled water at 4 °C for short term storage and in 15% glycerol at −20 °C for long term storage.

### 2.7. DNA Extraction, PCR, Sequencing, and Identification of Microbes

Fungal and yeast isolates (including those in co-culture) were grown on PDA supplemented with 50 mg/L each of streptomycin and tetracycline at 25 °C in the dark for two days (for fast-growing isolates) and up to five days (for slower-growing isolates). Total genomic DNA (gDNA) from fungal isolates was extracted using a MoBio PowerSoil^®^ DNA extraction kit (Mo-Bio Laboratories, Carlsbad, CA, USA) according to the manufacturer’s protocol. DNA extracts were diluted 1:4, and this served as the working DNA concentration for polymerase chain reaction (PCR) amplification. The ITS rDNA gene region (expected PCR product size ~650 bp) was amplified by PCR using universal primer pair ITS5/4 [61]. PCR reaction conditions consisted of an initial denaturation of 5 min at 94 °C, followed by 35 cycles of 1 min of denaturation at 94 °C, 1 min of annealing at 55 °C, 1 min primer extension at 72 °C, followed by a final extension of 5 min at 72 °C.

Bacterial isolates (pure isolates and those in co-culture) were grown on R2A supplemented with 50 mg/L each of streptomycin and tetracycline in the dark for 16 h or longer until growth was sufficient for extraction. Plates were flooded with 500–700 μL of TE buffer (10 mM Tris HCl, 1 mM EDTA, pH8; Sigma-Aldrich, St. Louis, MO, USA). The wash was collected and transferred to a 1.5 mL centrifuge tube, and 100 μL of 50 mg/L each of lysozyme and proteinase K (Sigma-Aldrich, St. Louis, MO, USA) was added. The samples were incubated at 37 °C for 2 h in a water bath, with occasional mixing by inversion. Immediately after incubation, the entire sample content was transferred to Maxwell^®^ 16 Cell DNA Purification kits (Promega, Madison, WI, USA) and gDNA was extracted according to the manufacturer’s protocol. DNA extracts were diluted 1:4, and this served as the working DNA concentration for PCR amplification. The 16S rRNA gene region (expected PCR product size ~1750 bp) was amplified by PCR with universal primer pairs 8F [62] and 1492R [63]. PCR conditions consisted of an initial denaturation of 5 min at 96 °C, followed by 33 cycles of 30 s of denaturation at 95 °C, 30 s of annealing at 55 °C, 2 min of primer extension at 72 °C, followed by a final extension of 5 min at 72 °C.

The PCR mixture (25 μL total volume) contained 12.5 μL of GoTaq^®^ Green Master Mix (Promega, Madison, WI, USA), 0.5 μL (10 μM) of each primer (Integrated DNA Technologies, Coralville, IA, USA), 6.5 μL of Nuclease-Free water (Promega, Madison, WI, USA), and 5 μL of DNA template. All PCRs were performed on a Thermal Cycler 2720 (Thermo Fisher Scientific, Bedford, MA, USA). PCR products were visualized on a 1.5% agarose gel stained with ethidium bromide (Sigma-Aldrich, St. Louis, MO, USA) and visualized under a MiniBIS Pro System (DNR Bio Imaging System, Neve Yamin, Israel). Where amplification failed, samples were processed once again from the first PCR. Samples producing amplicons were sent for purification and sequencing (MCLAB, San Francisco, CA, USA). Due to incorrect identifications and/or incomplete ITS sequences that were submitted to GenBank, additional approved markers for molecular identification through sequence comparisons of different housekeeping genes were required for accurate species identification, i.e., the β-tubulin (βTUB) gene for *Aspergillus* spp. [64] and translation elongation factor 1α (TEF1α) for *Fusarium* spp. [65,66,67]. Currently, there is no consensus about these supplementary barcodes since, for many taxa, they are genus-specific.

DNA sequences were manually edited using BioEdit software version 7.1.9 [68] to resolve nucleotide sequence ambiguities. Identification of sequences was performed using the BLASTn (Basic Local Alignment Search Tool) algorithm against the National Centre for Biotechnology Information (NCBI) GenBank database [69]. To confirm sequence identity, at least to the genus level, phylogenetic inference was carried out using the best fit nucleotide substitution model in MEGAX [70] using the maximum likelihood (ML) algorithm with 1000 bootstrapped replications. The trees were unrooted, and the 75% consensus trees (bs > 75%) are presented. Reference sequences are available as supplementary material.

### 2.8. Extracellular Lipase Assay

The enzyme activity of lipase was detected by using a modified Rhodamine agar plate method [71]. Briefly, each of the selected isolates was inoculated on nutrient agar (NA, HiMedia Laboratories LLC., West Chester, PA, USA) consisting of olive oil (3% *v*/*v*) (Sigma-Aldrich, St. Louis, MO, USA), Rhodamine 6G solution (0.001% *w*/*v*) (Sigma-Aldrich, St. Louis, MO, USA), pH 7, supplemented with 50 mg/L each of streptomycin and tetracycline and incubated at 25 °C in the dark. A 4 mm^3^ block from the advancing edge of a colony from an actively growing axenic culture was used for inoculation. As a control, uninoculated plates were also prepared. Assays were performed in triplicate and repeated. This assay was also carried out on plates with crude oil (1% *v*/*v*). The plates were examined under UV light, and activity was determined by visual inspection for yellow- to orange-colored fluorescence [71,72,73].

### 2.9. Statistical Analyses

Mean growth rates and the percentage of growth inhibition of all isolates on oil-amended media were analyzed using Minitab (version 17, Minitab LLC, Pennsylvania State University, University Park, PA, USA). Percentage growth inhibition values were arcsine-transformed prior to analysis. The least significant difference (LSD) test was used to separate means that were statistically significant at *p* ≤ 0.05.

The RStudio software version 1.3.1093 [74] statistical programming environment was used to perform statistical analyses. Abundance statistics, standardization (conversion to relative abundances), and patterns via rank-abundance dominance (RAD) analysis were performed using the package ‘BiodiversityR’, where the best fitting model was chosen by applying the Akaike information criterion (AIC), as described by Johnson and Omland [75]. Alpha diversity metrics [76] were generated using the package ‘BiodiversityR’, the Chao 1 richness estimator [77] and absolute dominance and Simpson’s dominance (D_2_) [78] indices using the package ‘microbiome’, and Simpson’s evenness (E_1/D_) [76] using the package ‘codyn’. Nonmetric multidimensional scaling (NMDS) ordination and ward clustering based on the Bray-Curtis dissimilarity matrix were used to analyze site differences based on the abundance of microbes and extrinsic factors. Adequacy of NMDS representation was assessed by simulation of Shepard’s plot and the goodness of fit plot. A similarity percentage (SIMPER) was used to identify the major contributing genera associated. One-way analysis of similarity (ANOSIM) was also carried out to determine the level of statistical variation between locations. Figures were produced using the packages ‘ggplot2’, ‘reshape’, ‘scales’, ‘viridisLite’, ‘viridis’, and ‘extrafont’, and package ‘dendextend’ was used to construct dendrograms. Statistical differences were calculated using one-way analysis of variance (ANOVA).

## 3. Results

### 3.1. Isolate Recovery

A total of 358 microbes were isolated from the eight sites (Table 2). From site P, 29 isolates were obtained; in Fyzabad, 128 isolates were obtained from F1 and 33 isolates from F2; from Vance, 16 isolates were obtained from V1, 17 isolates from V2, 52 isolates from V3, 33 isolates from V4, and 50 isolates from V5 (Table 2). These isolates included filamentous fungi, yeast, and coisolated bacteria, which were identified using sequence comparisons.

### 3.2. Isolation of Microbes from Contaminated Sites

All isolates were tested for their ability to grow in the presence of crude oil as a carbon source. From the total of 358 isolates recovered, 161 isolates demonstrated the capability to grow on 2% crude-oil-amended media (BHA, PDA, and R2A) and were retained in the final dataset (Table 2). Of the 161 isolates, 128 filamentous fungi and 33 bacterial co-cultures consisting of fungal–bacterial and yeast–bacterial co-cultures grew on oil and were proven to be crude oil utilizers based on their significant survival and growth in crude-oil-enriched conditions. The bacteria recovered were resistant to streptomycin and tetracycline (50 mg/L each), were present in co-culture with fungi/yeast, and performed well on oil-amended media. Thus, they were included in the study despite the intended selection of pure filamentous fungi.

### 3.3. Identification of Microbes with Demonstrated Oil-Degrading Capability in Vitro

**Figure 2:**
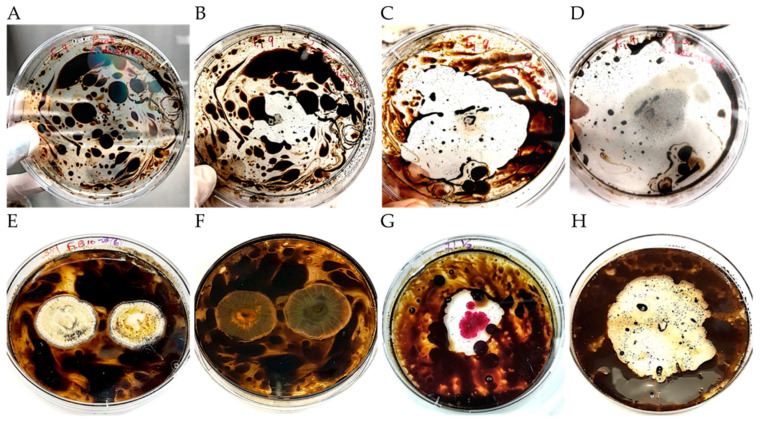
Bioassay on crude oil-amended plates. Examples of oil-degradation/utilization for (**A**) *Lecythophora* sp. (yeast) at 0 h; (**B**) *Lecythophora* sp. at 24 h; (**C**) *Lecythophora* sp. at 72 h; (**D**) *Lecythophora* sp. at 120 h; (**E**) *Aspergillus* sp. (filamentous fungus) front view at 7 days; (**F**) *Aspergillus* sp. with the disappearance of oil seen on the reverse view of the colony at 7 days; (**G**) *Serratia* sp. (bacterium) at 72 h; (**H**) *Janthinobacterium* sp. (bacterium) at 72 h.

Growth rates (diameter/mm/day) and visual observations of performance were recorded for each isolate (Figure 2, Tables S1 and S2). For fungal isolates grown on PDA and BHA with 2% crude oil, Fisher’s pairwise comparisons revealed no significant difference in growth on PDA and BHA (Table 3). The result indicated that all microbes recovered were able to utilize crude oil on both media types in vitro.

ITS and 16S sequence comparisons revealed the identities of the isolates in this final dataset (GenBank Accession Nos. MW633287 to MW633318 and MW670464 to MW670580). The identities of the Trinidad isolates were confirmed at the genus level for all sequences and at the species level for the majority of the sequences (Tables S3–S5). The microbes identified were compared to other fungi and bacteria previously identified in the literature for Trinidad. Of all the genera detected, only four were detected in Trinidad in previous studies (Table S6). A global check revealed that novel oil-degrading microbes, including *Oudemansiella* sp. and *Paraconiothyrium* sp., were, for the first time, shown as utilizers of petroleum hydrocarbons, and *Chaetomella* sp., *Neoascochyta* sp., *Sydowia* sp., *Lecythophora* sp., and *Sakaguchia* sp. were isolated from crude-oil-contaminated soil for the first time in Trinidad and globally.

### 3.4. Phylogenetic Analyses of Oil-Degrading Microbes

**Figure 3:**
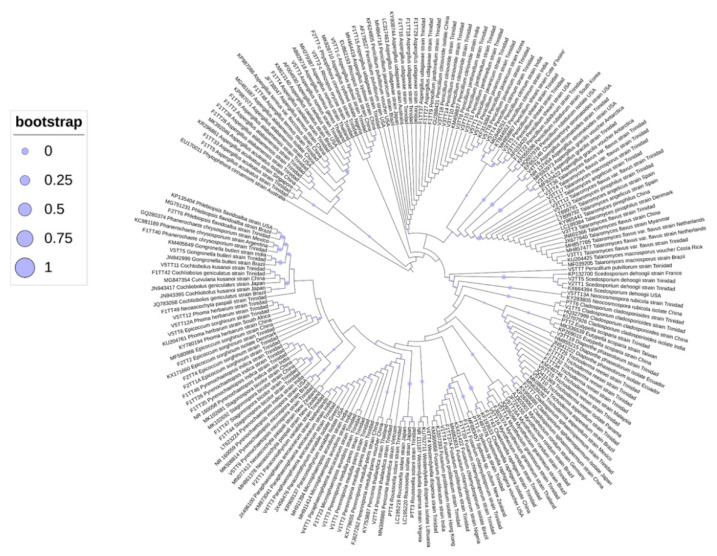
Unrooted ML phylogenetic tree for filamentous fungi.

**Figure 4:**
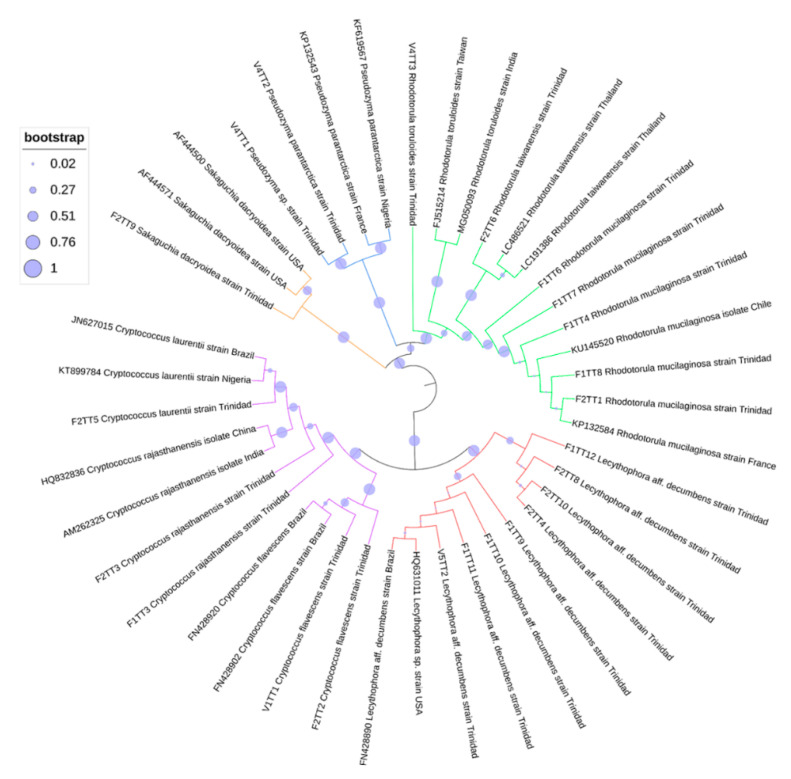
Unrooted ML phylogenetic tree for yeast.

**Figure 5:**
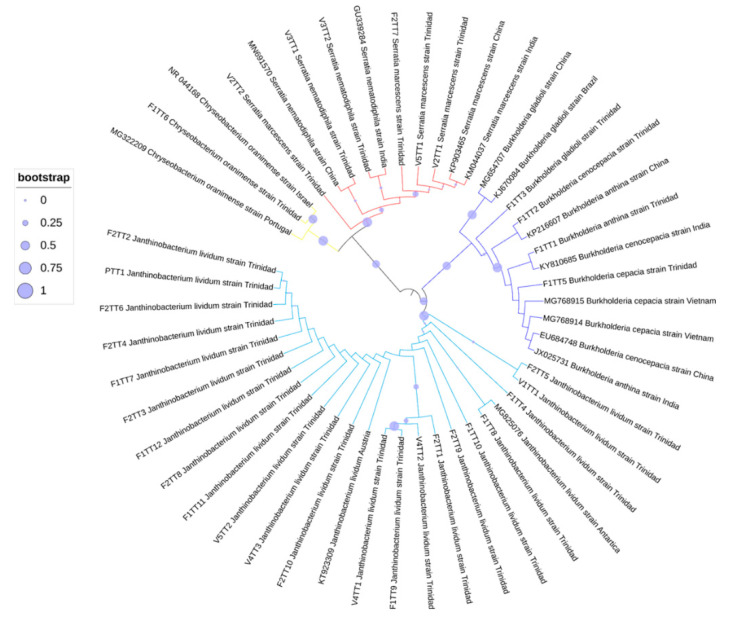
Unrooted ML phylogenetic tree for co-isolated bacteria based on 16S sequence comparisons.

Identification of microbes was based on ITS sequence comparisons with cognate sequences available in the GenBank database. Additional markers supported the identities indicated by ITS sequences, and, as such, unrooted phylogenetic trees were constructed based on ITS sequences using the inference of maximum likelihood with 1000 bootstrapped replicates (Figure 3, Figure 4 and Figure 5). The best fit model was also used to determine the placement of taxa, and the 50% consensus trees are presented. In each alignment, taxa were positioned according to genus, with high bootstrap support (bs > 75%), and the phylogenetic placement confirmed the identities of the isolated microbes.

### 3.5. Extracellular Lipase Production

**Figure 6:**
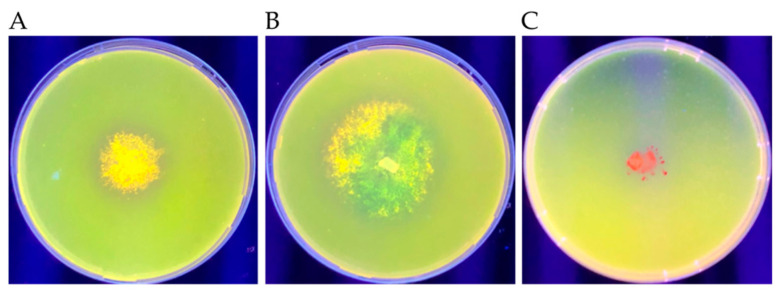
Lipase assay with Rhodamine 6G under UV light. (**A**,**B**) Yellow- to orange-colored fluorescence as a positive result. (**C**) Growth with no fluorescence as a negative result.

Microbes with the highest oil-degrading ability based on the parameters outlined in the previous section were selected for this extracellular lipase production assay. They were selected to determine whether the secreted lipase activity formed part of their mode(s) of oil utilization. After seven days of incubation on Rhodamine 6G plates at 25 °C, the presence of a yellow- to orange-colored fluorescence under UV light indicated secreted lipase activity. The fluorescence obtained for each isolate is described in Table 4, and examples of positive and negative results are shown in Figure 6.

### 3.6. Fungi and Yeast Composition Among all Sites

Of the 119 isolates that fulfilled the selection criteria, 96 filamentous fungal isolates were detected and 23 yeast isolates were recovered with proven oil-degrading capability in vitro. The culturable oil-degrading microbial communities were microbes identified in each site, and their composition and abundance were recorded and ranked in Table 5 and Table 6. The composition and absolute abundance of genera were highly heterogeneous (Figure S1, Table 5). The overall abundance of fungi and yeast was 80.67% and 19.33%, respectively.

**Figure 7:**
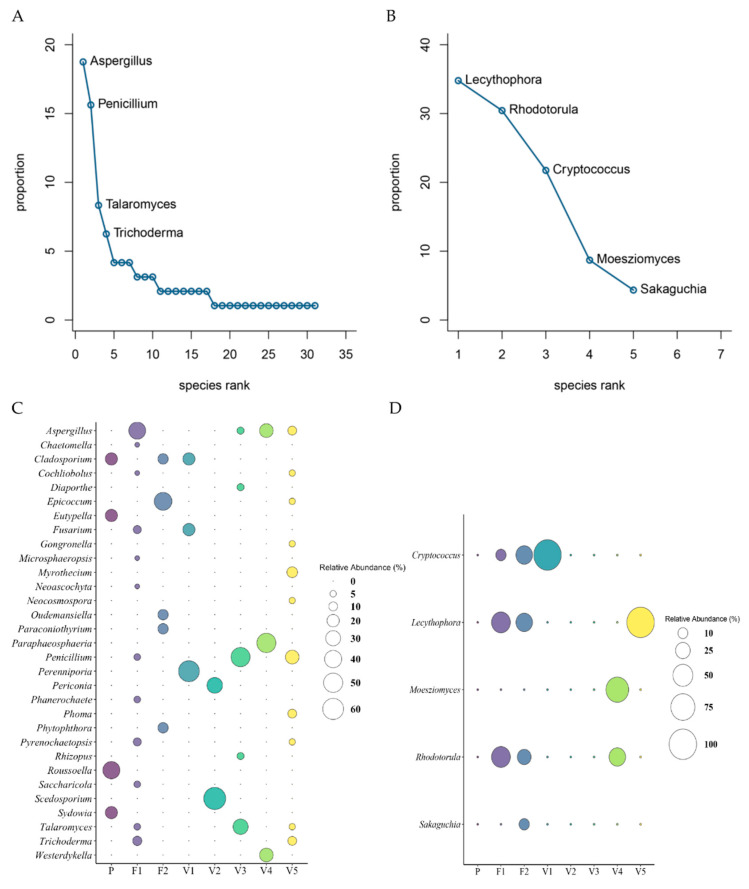
Rank abundance curves of (**A**) filamentous fungi and (**B**) yeast genus distributions are illustrated. The *y*-axis shows the proportional abundance (species abundance/total abundance) for a given genus, and the rank of genera from highest to lowest are represented on the *x*-axis. In the case of (**A**), several genera are dominant and appear written on the graph, while the long tail represents several rare genera in the fungal community. In the case of (**B**), the graph has no tail and, thus, shows that only a couple genera are rare. Based on the slopes observed, the filamentous fungal curve, which is steep, indicates lower evenness (i.e., a less homogeneous community is in terms of the abundances of genera present) than the yeast curve, which is less steep. The relative abundance of (**C**) filamentous fungi and (**D**) yeast genera is illustrated. In (**C**), 31 filamentous fungal genera were detected among all the sites; (**D**) 5 yeast genera were observed in 5 of the 8 sites.

The filamentous fungi identified belonged to 4 phyla, 7 classes, and 31 genera. The Ascomycota (90.63%) phylum dominated the culturable diversity. Other isolates belonged to the phyla Basidiomycota (6.25%), Mucoromycota (2.08%), and Oomycota (1.04%). At a class level, Eurotiomycetes dominated (46.88%) the dataset; Dothideomycetes (28.13%) and Sordariomycetes (14.58%) were detected, while Agaricomycetes, Mucoromycetes, Leotiomycetes, and Oomycetes were present in smaller numbers (6.25%, 2.08%, 1.04%, and 1.04%, respectively). The culturable fungal genera were dominated by *Aspergillus* and *Penicillium*, accounting for 18.75% (18 individuals) and 15.63% (15 individuals) of the entire fungal community, respectively. The less abundant genera identified were *Chaetomella*, *Cladosporium*, *Cochliobolus*, *Diaporthe*, *Epicoccum*, *Eutypella*, *Fusarium*, *Gongronella*, *Microsphaeropsis*, *Myrothecium*, *Neoascochyta*, *Neocosmospora*, *Oudemansiella*, *Paraconiothyrium*, *Paraphaeosphaeria*, *Perenniporia*, *Periconia*, *Phanerochaete*, *Phoma*, *Phytophthora*, *Pyrenochaetopsis*, *Rhizopus*, *Roussoella*, *Saccharicola*, *Scedosporium*, *Sydowia*, *Talaromyces*, *Trichoderma*, and *Westerdykella*, accounting for 1.04–8.33% (1 to 8 individuals) of the dataset. The abundance of the different fungal classes and genera detected was significantly different (α = 0). All data are presented in Table 6 and Figure 7.

The yeast recovered belonged to 2 phyla, Basidiomycota (65.23%) and Ascomycota (34.78%). At the class level, 6 taxa were identified. Sordariomycetes, Microbotryomycetes, Tremellomycetes, Ustilaginomycetes, Agaricomycetes, and Cystobasidiomycetes were detected (34.78%, 30.44%, 17.39%, 8.70%, 4.35%, and 4.35%, respectively). A total of 5 genera of yeast were identified. *Lecythophora* 34.78% (8 individuals), *Rhodotorula* 30.43% (7 individuals), and *Cryptococcus* 21.74% (5 individuals) were the more dominant genera detected. *Moesziomyces* and *Sakaguchia* accounted for 8.7% (2 individuals) and 4.35% (1 individual), respectively, of the yeast dataset. There was a highly significant difference in abundance of classes and genera found (α = 0.001). All data are presented in Table 6 and Figure 7.

The RAD or dominance/diversity plot [79,80] for filamentous fungal distribution was fitted to the lognormal Zipf model (Figure S2A). Lognormal communities are common in nature, especially in tropical regions where many factors act together and environmental factors keep some stability, thus representing a more balanced community. This was seen in the fungal abundance data, where many genera were present in very close abundance values, except for the two highly abundant genera, *Aspergillus* and *Penicillium*. The yeast community RAD plot showed the best fit to be the broken stick model, which suggests that individuals are randomly distributed among the observed genera and that there are no fitted parameters (Figure S2B). Broken stick distribution usually applies to small and homogenous communities, while lognormal distribution applies to large and heterogeneous communities [81]. This common trend was reflected in this study, as represented by the smaller total abundance for yeast versus filamentous fungi as well as the number of different genera detected for filamentous fungi versus yeast. The AIC values for the RAD plots can be found in Table S7.

### 3.7. Microbial Composition of Fungi and Yeast per Site

In this study, we used the relative abundance, which is the percent composition of a given microbe relative to the total number of microbes in the site, to examine the abundances of the genera detected. The composition and abundance of genera were highly heterogeneous in each site, even for samples retrieved from the same locality, and the dominant genera in each site are highlighted in Table 5. The relative abundances of filamentous fungi and yeast in the 8 sites can be seen in Figure 7 and Table S8.

For the filamentous fungal isolates, Fisher’s pairwise comparisons revealed that there is a significant difference in the abundance of fungi in F1 and V5 compared to the other sites. For the yeast isolates, Fisher’s pairwise comparisons revealed that there was a significant difference in the abundance of yeast, where sites F1, F2, and V5 were significantly different from sites P, V1, V2, and V3. There was no detection of yeast in three sites (P, V2, and V3). Fisher’s pairwise comparison data can be found in Table 3.

### 3.8. Diversity of Fungi and Yeast

The filamentous fungi and yeast community α-diversity estimates, including the Shannon’s and Simpson’s indices, exhibited associations among the sites. In the fungal community, two sites, F1 and V5, grouped together by Fisher’s pairwise comparisons (Table 3), were the most diverse (F1: H = 2.07, D_1_ = 0.81; V5: H = 2.22, D_1_ = 0.87). These sites were multispecies communities (F1: S = 12; V5: S = 11). Site V2 was the least diverse (H = 0.64, D_1_ = 0.44) and was the sparsest community, consisting of only two species (S = 2). The diversity between the sites was highly significantly different (α = 0). Overall, the α-diversity estimates revealed that Vance was the most diverse location (S = 19, H = 2.52, D_1_ = 0.88), closely followed by Fyzabad (S = 17, H = 2.41, D_1_ = 0.86), while Pablito was the least diverse (S = 4, H = 1.33, D_1_ = 0.72). In all cases, the Chao 1 richness estimator indicated that the theoretical richness for each site was more than the observed richness. All diversity indices for the filamentous fungal community are given in Table S9.

In the yeast community, F1 and F2 were grouped in Fisher’s pairwise comparisons (Table 3) and were the most diverse (F1: H = 0.97, D_1_ = 0.60; F2: H = 1.31, D_1_ = 0.72), having the most species variation (F1: S = 3; F2: S = 4). Sites V1 and V5 were single-species communities and, thus, lacked diversity (H = 0, D_1_ = 0). Overall, Vance was the more diverse location (H = 1.33, D_1_ = 0.72) compared to Fyzabad (H = 1.22, D_1_ = 0.69). The Chao 1 richness estimator for Fyzabad showed that the observed richness equaled the theoretical richness; however, Vance had a lower observed richness compared to the theoretical one. All diversity indices for the yeast community are shown in Table S10.

### 3.9. Community Ordination and Clustering

**Figure 8:**
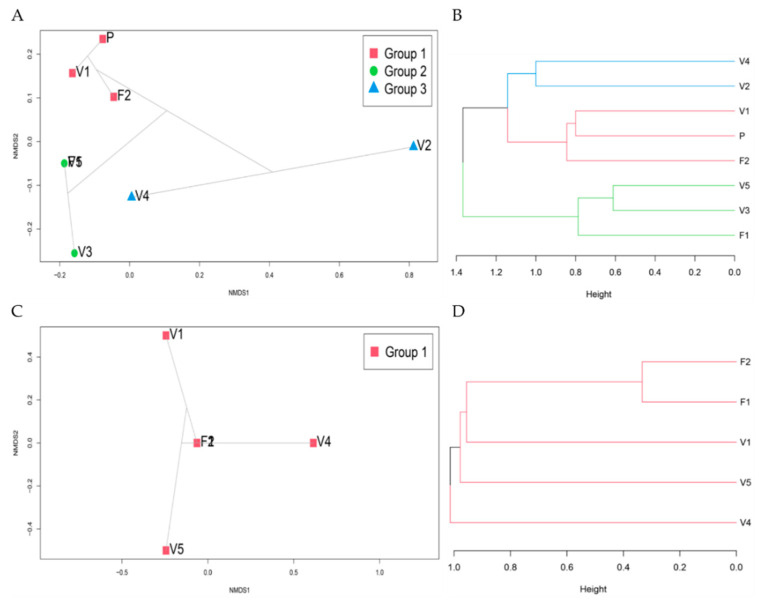
NMDS ordination plots based on the Bray-Curtis distances and ward cluster dendrograms. (**A**): NMDS plot of the filamentous fungal community; note that V5 and F1 are stacked together. (**B**,**D**) Ward cluster of filamentous fungal and yeast community, respectively. (**C**) NMDS plot of yeast community; note that F1 and F2 are stacked together.

**Figure 9:**
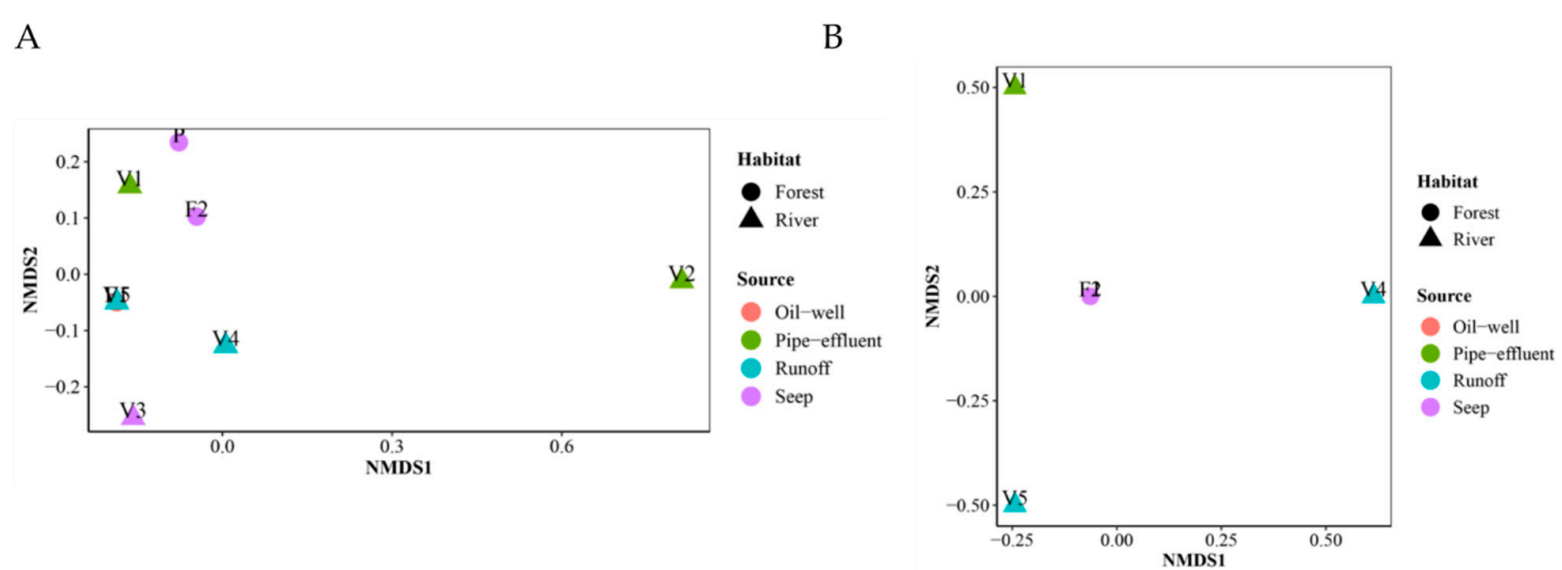
NMDS ordination plots based on the Bray-Curtis distances for environmental factors habitat and source. (**A**) NMDS plot of the filamentous fungal community; note that V5 and F1 are stacked together, where the V5 symbol is a blue triangle and F1 is a red circle. (**B**) NMDS plot of the yeast community; note that F1 and F2 are stacked together, where F1 is a red circle and F2 is a purple circle.

Through further investigation into the distribution of the sites’ microbial communities, the NMDS and ward clustering performed indicated that distinct microbial communities inhabited each site. RAD lognormal fungal distribution suggested large and heterogeneous communities (recall abundance of 96 filamentous fungi belonging to 31 genera was detected, with varying abundances and occurrences in each site), and broken stick yeast distribution suggested small homogenous communities (abundance of 23 yeast belonging to 5 genera was detected, with more homogenous occurrences among the sites). NMDs and Ward clustering confirmed this, as indicated by the order in which the clusters are joined and the variation in heights between these clusters (Figure 8). Adequacy of the NMDS representation was assessed by the low stress values obtained (<0.01) and the simulation of Shepard’s plot and the goodness of fit plot (Figure S3). SIMPER analysis indicated that specific genera were driving the differences between the sites (Table S11). ANOSIM analysis conveyed that similarities between locations are higher than within them but with no significant difference (Figure S4). There was no distinction in the filamentous fungal and yeast communities in the various sites based on the habitat type or source, apart from sites F1 and F2 in the yeast community (Figure 9).

## 4. Discussion

Indigenous microorganisms become highly adapted to survival in hydrocarbon-contaminated terrestrial environments through selective enrichment and genetic modifications that enable them to catabolize xenobiotic chemicals [82,83,84,85,86]. The potential of indigenous bacteria to degrade petroleum hydrocarbons isolated from Pitch Lake at La Brea in Trinidad has been reported [87,88,89,90]. Ramoutar et al. [91] described fungi isolated from Pitch Lake, including *Aspergillus*, *Fusarium*, *Penicillium*, *Curvularia*, and *Mucorales*, that were capable of PAH-degradation rates of 10–65% for single isolates and 64–76% for select consortia. Microbial populations indigenous to the many other naturally occurring hydrocarbon seeps and chronically polluted areas in Trinidad should, therefore, be more fully explored for their bioremediation and industrial potential. Stefani et al. [35] concluded that effective bioremediation remains a function of those microbes that are indigenous to a given polluted site. This investigation focused on eight such sites located in the southern peninsula of the island, and our findings indicate novel oil-degrading filamentous fungi and yeast as single-isolate degraders and naturally occurring consortia, with specific bacterial species not previously reported in the literature. Because of the different sources and means of identification of oil-degrading strains and isolates reported in the literature, the terms ‘strains’ and ‘isolates’ are used interchangeably in comparison to our isolates to retain the accuracy of the original reporting. A comparison of species reported in the literature with those isolated in this study revealed similarities in microbial composition; however, novel indigenous species with demonstrated oil-degrading ability were also revealed.

### 4.1. Fungi

The fungal community in the oil-contaminated soils consisted of species belonging to four phyla, where Ascomycota dominated (90.63%), followed by Basidiomycota (6.25%). The dominance of Ascomycota, followed by Basidiomycota, in polluted soils has been acknowledged: Siles and Margesin [92] reported that of the 87 operational taxonomic units (OTUs) that comprised the fungal community, up to 95.3% of the classified sequences belonged to the Ascomycota phylum and up to 62.7% belonged to the Basidiomycota phylum; Spini et al. [93] reported that of 94 unique fungal colonies, 95% belonged to Ascomycota and only 5% to Basidiomycota. PAHs include a group of priority pollutants that pose a serious threat to the health of humans and ecosystems [18]. Multiple reviews have highlighted the biotransformation of PAHs with Ascomycota and Basidiomycota fungi [43,94,95].

The fungal community was affiliated with 31 fungal genera, including *Aspergillus* (18.75%), *Penicillium* (15.63%), *Talaromyces* (8.33%), *Trichoderma* (6.25%), *Epicoccum*, *Fusarium*, *Pyrenochaetopsis*, *Cladosporium*, *Myrothecium*, *Perenniporia*, *Cochliobolus*, *Paraphaeosphaeria*, *Phanerochaete*, *Phoma*, *Roussoella*, *Saccharicola*, *Scedosporium*, *Chaetomella*, *Diaporthe*, *Eutypella*, *Gongronella*, *Microsphaeropsis*, *Neoascochyta*, *Neocosmospora*, *Oudemansiella*, *Paraconiothyrium*, *Periconia*, *Phytophthora*, *Rhizopus*, *Sydowia*, and *Westerdykella*, which were less abundant and represented 1% to 4% of the recovered isolates.

Hydrocarbon-degrading strains of *Aspergillus* and *Penicillium* are frequently isolated from soil [6]. In a recent study, *Aspergillus* and *Trichoderma* were reported as the most abundant genera in soils polluted with mixtures of aliphatic and polycyclic hydrocarbons [93], and, more recently, *Aspergillus*, *Penicillium*, and *Trichoderma* were among the culturable petroleum-degrading fungi isolated from soil samples [96]. *Aspergillus* and *Penicillium* have been reported as remediation agents of PAHs, using enzymes such as oxygenases during transformation [95,97,98].

Ligninolytic fungi such as *Phanerochaete chrysosporium* are well-known degraders of PAHs, including benzo[a]pyrene, due to the secretion of oxidative enzymes [99]. Members of the genus *Cladosporium*, *Fusarium*, *Scedosporium*, *Eutypella*, *Talaromyces*, and *Cochliobolus* [93,96,100,101,102], isolated from a variety of oil-contaminated sources, are also well-known biodegraders that are capable of metabolizing a range of compounds. Morales et al. [103] characterized a strain of *Scedosporium* that could degrade several petroleum hydrocarbons and identified 11,195 protein-coding genes that may be involved in hydrocarbon degradation pathways.

With respect to rarer genera, *Epicoccum*, *Pyrenochaetopsis*, *Rhizopus*, and *Phoma* have been reported as oil-degrading microbes [104,105,106]. *Myrothecium*, as a petroleum-utilizing microbe [107], has also been found to immobilize toxic metals, which may be relevant to specific bioremediation strategies as these fungi are capable of mediating metal precipitation [108]. *Paraphaeosphaeria* has been isolated from an asphalt seep but not investigated for its bioremediation potential [109]. *Roussoella* isolated from contaminated soil grows in the presence of toluene, hexadecane, and polychlorinated biphenyls (PCB) and utilizes ligninolytic enzymes for wood degradation [110]. *Perenniporia* [111], *Saccharicola*, and *Diaporthe* were reported as petroleum hydrocarbon degraders [112] with potential bioremediation functions, but the latter strains were isolated from leaf and stem tissues from plants that were growing in soil contaminated with crude oil and were not isolated from the contaminated soil itself [113]. *Gongronella* is capable of degrading hydrocarbons [114], including pyrene [115]. *Microsphaeropsis* is a promising genus for microbe-assisted phytoremediation [116,117]. *Microsphaeropsis* and *Westerdykella* exhibit the ability to degrade poly-ethylene terephthalate (PET depolymerization), and have shown upregulated expression of lipase and esterase activities [118]. *Westerdykella* also utilizes polycyclic PAHs [119] with tolerance to pyrene [120] and the depletion of fluorene [121]. *Periconia*, which is isolated from oil-contaminated soil, grows on petrol and kerosene, and can degrade oil [122], is one of the many microbes detected in soil for a phytoremediation strategy to remediate crude-oil-polluted soil [123]; it has also been isolated from a marine site with frequent oil spills but not investigated further for crude oil degradative capabilities [124]. *Phytophthora* has been reported as an aromatic degrader [125], specifically for naphthalene [126], and *Neocosmospora* isolated from contaminated soil has demonstrated hydrocarbonoclastic abilities [127].

Rarely isolated genera such as *Oudemansiella* has shown enzymatic activity similar to that of oil-degrading microbes, which also aids in its ability to decolorize polymeric dyes and can be correlated with xenobiotic degradation, just as petroleum hydrocarbon molecules are similar to lignin molecules [128]. It has also been used to promote the dissipation of pyrene from soil [129]. *Paraconiothyrium* has been isolated from mangroves that are impacted by PAH contamination [130]. In this study, *Oudemansiella* and *Paraconiothyrium* were, for the first time, shown to be utilizers of petroleum hydrocarbons. Additionally, new oil-degrading fungi, *Chaetomella*, *Neoascochyta*, and *Sydowia*, were isolated from contaminated soil and have not been reported elsewhere in any known bioremediation study. Since these fungi degrade crude oil, the possibility for their use exists in the development of microbial technology for the remediation of crude-oil-contaminated sites.

### 4.2. Yeast

All yeast isolates were affiliated to Basidiomycota (65.23%) and Ascomycota (34.78%) phyla, which is the most reported taxonomic phylum in terrestrial environments (see [131,132]). The yeast detected in this study belonged to the genera *Lecythophora* (34.78%), *Rhodotorula* (30.45%), *Cryptococcus* (21.74%), *Moesziomyces* (synonym of the asexually typified genus *Pseudozyma*) (8.70%), and *Sakaguchia* (4.35%).

*Rhodotorula* is a commonly isolated yeast from oil-contaminated environments [6,133]. In a recent study by Mikolasch et al. [134], *Rhodotorula* was isolated from oil-contaminated soils using a variety of hydrocarbons and could break down cyclohexanone. In a study by Hashem et al. [135] of the 67 yeast strains tested, *Rhodotorula* was among the top six isolates for its ability to degrade both aliphatic and aromatic hydrocarbons, and further investigation revealed two strains as the best degraders of octane and pyrene, with other research reporting similar findings [136]. Species of *Rhodotorula* have also been shown as degraders of decane and nonane [137]. *Rhodotorula* and *Cryptococcus* were found to catabolize benzene compounds [131] and are potent degraders of diesel oil, utilizing a range of enzymes including aminopyrine-N-demethylase, alcohol dehydrogenase, aldehyde dehydrogenase, catalase, and glutathione S transferase. The cytochrome P450 monooxygenases were especially important to diesel oil degradation [138], and isolates of these genera from petroleum-contaminated environments could produce secreted lipases [133].

The yeast *Moesziomyces* (previously cited as *Pseudozyma* but now reassigned based on molecular phylogenetic analysis [139,140] has rarely been described as hydrocarbon-degrading but was recently shown to be efficient tetradecane- [134] and diesel-fuel-degraders [141]. Furthermore, the yeast *Lecythophora* and *Sakaguchia* have, to our knowledge, never been reported in the literature as known oil degraders isolated from oil-contaminated soils. *Lecythophora* species have been shown to degrade PAHs such as pyrene [142,143]. *Sakaguchia* has been previously isolated from a marine environment, and it was surmised that its coexistence with another oil-degrading yeast species suggests hydrocarbon utilization capability [144].

### 4.3. Co-isolated Bacteria

At the genus level, *Janthinobacterium* (66.67%), *Serratia* (18.18%), *Burkholderia* (12.12%), and *Chryseobacterium* (3.03%) were isolated from crude-oil-contaminated soil in this study and had a similar occurrence with previously reported hydrocarbon-degrading members of the same genera [46,93,145,146,147]. Since only a few bacteria can grow on high molecular weight PAHs and the metabolism of PAHs by bacteria is limited due to the poor bioavailability of these compounds, the importance of research on bacteria capable of degrading petroleum hydrocarbons, especially PAHs, has been highlighted [43]. In addition, there is a lack of degradation studies on the complexity of PAHs present in multicomponent mixtures in natural environments, which influences the rate and extent of biodegradation [84]. In agreement with a previous study [148], the dominant bacterium isolated in this study was *Janthinobacterium*. *Janthinobacterium* has been isolated from oil-contaminated soil [148] and can degrade carbazole [149] and persistent organic pollutants, explicitly polychlorinated biphenyls (PCBs) [150]. *Chryseobacterium* has been isolated from oil-contaminated soil [145], and its use in consortia has shown potential for its application in the bioremediation of soils [151], including PAHs [152]. *Serratia* has shown a relatively high capacity to degrade a wide spectrum of hydrocarbons [93,146,153] and demonstrated degradation rates of up to 80.4% for benzo[a]pyrene [154]. *Burkholderia* has been reported as one of the limited taxonomic groups that can degrade PAHs in soil [46,147] and via bacterial consortium [155] after engaging aromatic degradation pathways [156]. *Pseudomonas*, one of the most popular and dominant bacteria, was, surprisingly, not isolated in this study [147].

### 4.4. Extracellular Lipase Production

Extracellular microbial lipases and their wide range of applications represent commercial value for such microbes [157,158,159]. Enhanced biodegradation of petroleum hydrocarbons is maintained by biocatalysts such as lipases [158]. Among the seven isolates screened, the most promising lipase producer appeared to be F1 40: *Phanerochaete chrysosporium*. This species is well-documented for its ability to produce lipase and its role in degrading xenobiotics, including PAHs [99,160]. Isolates V2 5 (*Scedosporium dehoogii*), F1 1 (*Burkholderia anthina*), V2 1 (*Serratia marcescens*), and F1 9 (*Lecythophora aff. decumbens*) also showed lipase production but to a lesser extent compared with *P. chrysosporium*. Two isolates, F1 6 (*Chryseobacterium oranimense*) and F1 7 (*Rhodotorula mucilaginosa*), showed no secreted lipase production. The media composition and physiochemical factors (temperature, pH, and dissolved oxygen) can influence the production of extracellular lipases [158,161]. Lipases are inducible enzymes, and one of the most influential factors is the carbon source [161,162]. Further studies on the induction of extracellular lipase activity are warranted.

### 4.5. Novel Oil-Degraders from Trinidad

This is the first report of representatives of fungal genera *Penicillium*, *Talaromyces*, *Trichoderma*, *Epicoccum*, *Pyrenochaetopsis*, *Cladosporium*, *Myrothecium*, *Perenniporia*, *Cochliobolus*, *Paraphaeosphaeria*, *Phanerochaete*, *Phoma*, *Roussoella*, *Saccharicola*, *Scedosporium*, *Chaetomella*, *Diaporthe*, *Eutypella*, *Gongronella*, *Microsphaeropsis*, *Neoascochyta*, *Neocosmospora*, *Oudemansiella*, *Paraconiothyrium*, *Periconia*, *Phytophthora*, *Rhizopus*, *Sydowia*, and *Westerdykella*; bacterial genera *Janthinobacterium*, *Serratia*, and *Chryseobacterium*; and yeast genera *Lecythophora*, *Rhodotorula*, *Cryptococcus*, *Moesziomyces*, and *Sakaguchia* in Trinidad with demonstrated hydrocarbon-degrading ability in vitro. This finding is especially important due to the deficit of published data on the use of common commercial products in tropical environments and their effectiveness and suitability in local conditions [87]. Such indigenous microorganisms, which have been established and developed through selective enrichment and genetic modifications to survive and thrive in their hydrocarbon-polluted environment [163], can serve as a more appropriate product source compared to other commercial counterparts [87].

Methods of detection are critical to the analysis of microbial diversity. Utilization of culture-dependent and culture-independent approaches can provide two exceedingly different microbial compositions [40]. While culture-based methods are the classical approach to isolated hydrocarbon-degrading microbes, it must be noted that less than 1% of the microbes present in soil are cultivable [164]. However, understanding the contribution of these microbes to the complex and cooperative processes of survival in chronically contaminated soil requires their cultivation [165]. This study was a culture-dependent one, and the results suggested the activation and growth of rarer species during culture. In a study by M’rassi et al. [37], 40% of the strains isolated from oil-contaminated soil could not be detected because microbial assemblages are influenced by several different factors [6,29]. The microenvironment, geographical location, reservoir temperatures, soil physico-chemical properties, and oil composition all affect the microbial community structure and abundance [29,164,166,167,168]. In accordance with previous research, the specific conditions of the culture-based approach for microbe recovery impacted the observed microbial diversity in this study and the detection of rarer taxa [29,40]. Culture-dependent methods can also elicit the favorable growth of microbes that are experiencing antagonistic effects and those that no longer have to compete for limited resources in their natural environment. Conversely, co-metabolism between fungi and yeast with bacteria, as found in this study, must also be considered and investigated [169].

The isolates in this study were identified by sequence comparisons and phylogenetic inference, and the results indicated that taxa placement was genus-specific in all cases, irrespective of the origin of the sequence, i.e., the sequence of an isolate or strain from a source that may or may not be contaminated with crude oil. Further studies on the mapping and profiling of single genes and biochemical gene clusters to PAH-degradative pathways of generalist oil degraders and those which may be specific to indigenous microbes in chronically contaminated soil that promote the enrichment of specialist oil degraders need to be carried out.

## 5. Conclusions

This study focused on the unique culturable microbial composition of soil chronically contaminated with crude oil. Several fungal and yeast species not previously reported have been identified here as potential crude oil degraders with demonstrated lipase production and secretion that aid in oil break-down. The latter aspect may also be important to several industrial applications. Co-metabolism between specific yeast and bacteria was also detected, which supports the consortium approach to understanding in situ degradation. These co-cultures were difficult to separate in vitro, and it is likely that survival is codependent in the natural environment. Biodiversity analysis indicated that specific fungal species of *Aspergillus* and *Penicillium* dominated the culturable microbial landscape, and these findings were similar to other studies. While yeast composition and species diversity are limited, the yeast–bacteria co-cultures demonstrated a very high potential for crude oil degradation. Cumulatively, the findings of this study are especially important for exploitation in tropical environments and for understanding the use of indigenous microbes in sustainable bioremediation and biodeterioration efforts.

## Figures and Tables

**Table 1:** Sampling data of the 8 geographically separated sites and the physical properties [58] of the 3 geographically separated localities in southwestern Trinidad.

Locality	Formation	Depth (ft)	Porosity (%)	Permeability (md)	Oil Gravity	Viscosity	Oil Saturation	Site Type
Pablito (P)	Morne L’Enfer	1200–1700	28	250	13–21	160–550	65–75	Forest
Fyzabad Forest Reserve (F)	Morne L’Enfer	1200–1701	28	250	13–22	160–550	65–75	Forest
Vance River (V)	Morne L’Enfer	1200–1702	28	250	13–23	160–550	65–75	River
**Site**	**Description**							
P1	Natural seep/mud volcano							
F1	Manmade seep—abandoned oil well							
F2	Natural oil seep							
V1	Heavily oiled soil—pipeline contamination							
V2	Heavily oiled soil—pipeline contamination							
V3	Heavily oiled soil—natural seep							
V4	Heavily oiled soil—effluent contamination							
V5	Heavily oiled soil—effluent contamination							

**Table 2:** Isolate recovery from the 8 sites in southwestern Trinidad. A total of 152 microbes were retained in the final dataset (96 filamentous fungi + 33 bacteria + 23 yeast).

Locality	Site	No. of Soil Microbes Recovered	Crude-Oil-Utilizing Microbes		Selected Microbes			
			Fungi	Bacteria Co-cultures	Fungi	Bacteria Co-cultures	Yeast *	Fungi *
Pablito (P)	P	29	6	1	4	1	-	1
Fyzabad (F)	F1	128	50	12	35	12	9	1
	F2	33	11	10	6	10	9	1
Vance (V)	V1	16	5	1	5	1	1	-
	V2	17	9	2	3	2	-	-
	V3	52	19	2	14	2	-	2
	V4	33	8	3	4	3	3	-
	V5	50	20	2	19	2	1	1
**Total**		**358**	**128**	**33**	**90**	**33**	**23/33**	**6/33**
Total			161 isolates		123 isolates 152 microbes		23	6

* The breakdown of the number of yeast and fungi that were in co-culture with a bacterial species.

**Table 3:** Minimum, maximum, mean absolute abundance, and Fisher’s pairwise comparison according to media type and sites.

	N	Mean	SE Mean	Std. Dev	Minimum	Maximum	Grouping *
**Media type**							
PDA	114	5.395	0.245	2.621	1.340	13.803	A
BHA	122	4.702	0.268	2.955	0.409	14.91	A
Fungi							
P	31	0.161	0.082	0.454	0	2	B
F1	31	1.161	0.473	2.634	0	14	A
F2	31	0.226	0.111	0.617	0	3	B
V1	31	0.161	0.105	0.583	0	3	B
V2	31	0.097	0.071	0.396	0	2	B
V3	31	0.516	0.300	1.671	0	8	B
V4	31	0.129	0.077	0.428	0	2	B
V5	31	0.645	0.205	1.142	0	5	A
Yeast							
P	5	0.000	0.000	0.000	0	0	B
F1	5	1.800	0.917	2.049	0	4	A
F2	5	1.800	0.583	1.304	0	3	A
V1	5	0.200	0.200	0.447	0	1	B
V2	5	0.000	0.000	0.000	0	0	B
V3	5	0.000	0.000	0.000	0	0	B
V4	5	0.600	0.400	0.894	0	2	A B
V5	5	0.200	0.200	0.447	0	1	A

* Fisher’s LSD method and 95% confidence; means that do not share the same letter are significantly different.

**Table 4:** Lipase activity of selected isolates cultured on olive oil and Rhodamine 6G agar plates.

Microbe	Isolate	Rhodamine Intensity *
Fungi	F1 40	+++
	V2 5	+
Bacteria	F1 1	++
	F1 6	-
	V2 1	++
Yeast	F1 7	-
	F1 9	+

* Fluorescence intensity: (+) low; (++) medium; (+++) high; (-) no lipase produced.

**Table 5:** Top crude-oil-degrading genera in the 8 sites in southwestern Trinidad.

Site	P	F1	F2	V1	V2	V3	V4	V5	Sum
*Aspergillus*	0	14	0	0	0	1▪	1	2▪	18
*Chaetomella*	0	1	0	0	0	0	0	0	1
*Cladosporium*	1	0	1	1	0	0	0	0	3
*Cochliobolus*	0	1	0	0	0	0	0	1	2
*Diaporthe*	0	0	0	0	0	1	0	0	1
*Epicoccum*	0	0	3	0	0	0	0	1	4
*** Eutypella	1	0	0	0	0	0	0	0	1
*Fusarium*	0	3	0	1	0	0	0	0	4
*Gongronella*	0	0	0	0	0	0	0	1	1
*Microsphaeropsis*	0	1	0	0	0	0	0	0	1
*Myrothecium*	0	0	0	0	0	0	0	3	3
*Neoascochyta*	0	1	0	0	0	0	0	0	1
*Neocosmospora*	0	0	0	0	0	0	0	1	1
*Oudemansiella*	0	0	1	0	0	0	0	0	1
*Paraconiothyrium*	0	0	1	0	0	0	0	0	1
*Paraphaeosphaeria*	0	0	0	0	0	0	2	0	2
*Penicillium*	0	2	0	0	0	8	0	5	15
*Perenniporia*	0	0	0	3	0	0	0	0	3
*Periconia*	0	0	0	0	1	0	0	0	1
*Phanerochaete*	0	2▪	0	0	0	0	0	0	2
*Phoma*	0	0	0	0	0	0	0	2	2
*Phytophthora*	0	0	1▪	0	0	0	0	0	1
*Pyrenochaetopsis*	0	3	0	0	0	0	0	1	4
*Rhizopus*	0	0	0	0	0	1▪	0	0	1
*** Roussoella	2	0	0	0	0	0	0	0	2
*Saccharicola*	0	2	0	0	0	0	0	0	2
*Scedosporium*	0	0	0	0	2	0	0	0	2
*** Sydowia	1▪	0	0	0	0	0	0	0	1
*Talaromyces*	0	2	0	0	0	5	0	1	8
*Trichoderma*	0	4	0	0	0	0	0	2	6
*Westerdykella*	0	0	0	0	0	0	1	0	1
**Fungi Sum**	**5**	**36**	**7**	**5**	**3**	**16**	**4**	**20**	**96**
*Cryptococcus*	0	1	3	1	0	0	0	0	5
* Lecythophora	0	4	3	0	0	0	0	1	8
*Moesziomyces*	0	0	0	0	0	0	2	0	2
•*Rhodotorula*	0	4	2	0	0	0	1	0	7
*Sakaguchia*	0	0	1	0	0	0	0	0	1
**Yeast Sum**	**0**	**9**	**9**	**1**	**0**	**0**	**3**	**1**	**23**
*Burkholderia*	0	4	0	0	0	0	0	0	4
*Chryseobacterium*	0	1	0	0	0	0	0	0	1
*Janthinobacterium*	1	7	9	1	0	0	3	1	22
*Serratia*	0	0	1	0	2	2	0	1	6
**Bacteria Sum**	**1**	**12**	**10**	**1**	**2**	**2**	**3**	**2**	**33**
**Total Microbial Sum**									**152**

▪ Represents an abundance of 1 for that genera existing in co-culture; • abundance of genera among localities is significant (α = 0.05; *p* ≤ 0.05); * abundance of genera among localities is highly significant (α = 0.01; *p* ≤ 0.01); *** abundance of genera among localities is extremely significant (α = 0). Shaded boxes represent the dominant genus (absolute dominance) in each site. Since filamentous fungi and yeast were selected for the study, bacteria were not subject to these statistics and are included here to show distribution among sites alone.

**Table 6:** Ranked abundance of fungi and yeast.

Taxonomy	Rank	A	%	Taxonomy	Rank	A	%
**PHYLA**				**GENUS**			
**Filamentous Fungi**				**Filamentous Fungi**		**96**	**80.672**
Ascomycota	1	87	90.625	*Aspergillus*	1	18	18.750
Basidiomycota	2	6	6.250	*Penicillium*	2	15	15.625
Mucoromycota	3	2	2.083	*Talaromyces*	3	8	8.333
Oomycota	4	1	1.042	*Trichoderma*	4	6	6.250
**Total**		96		*Epicoccum*	5	4	4.167
**ANOVA**		0.195		*Fusarium*	6	4	4.167
**Yeast**				*Pyrenochaetopsis*	7	4	4.167
Basidiomycota	1	15	65.217	*Cladosporium*	8	3	3.125
Ascomycota	2	8	34.783	*Myrothecium*	9	3	3.125
**Total**		23		*Perenniporia*	10	3	3.125
**CLASS**				*Cochliobolus*	11	2	2.083
**Filamentous Fungi**				*Paraphaeosphaeria*	12	2	2.083
Eurotiomycetes	1	45	46.875	*Phanerochaete*	13	2	2.083
Dothideomycetes	2	27	28.125	*Phoma*	14	2	2.083
Sordariomycetes	3	14	14.583	*Roussoella*	15	2	2.083
Agaricomycetes	4	6	6.250	*Saccharicola*	16	2	2.083
Mucoromycetes	5	2	2.083	*Scedosporium*	17	2	2.083
Oomycetes	6	1	1.042	*Chaetomella*	18	1	1.042
Leotiomycetes	7	1	1.042	*Diaporthe*	19	1	1.042
**Total**		96		*Eutypella*	20	1	1.042
**ANOVA**		0.005 **		*Gongronella*	21	1	1.042
**Yeast**				*Microsphaeropsis*	22	1	1.042
Sordariomycetes	1	8	34.783	*Neoascochyta*	23	1	1.042
Microbotryomycetes	2	7	30.435	*Neocosmospora*	24	1	1.042
Tremellomycetes	3	4	17.391	*Oudemansiella*	25	1	1.042
Ustilaginomycetes	4	2	8.696	*Paraconiothyrium*	26	1	1.042
Agaricomycetes	5	1	4.348	*Periconia*	27	1	1.042
Cystobasidiomycetes	6	1	4.348	*Phytophthora*	28	1	1.042
**Total**		23		*Rhizopus*	29	1	1.042
**ANOVA**		0.002 ***		*Sydowia*	30	1	1.042
				*Westerdykella*	31	1	1.042
				**Total**		96	100
				**ANOVA**	1.5 × 10^−5^ ***		
				**Yeast**		**23**	**19.328**
				*Lecythophora*	1	8	34.783
				*Rhodotorula*	2	7	30.435
				*Cryptococcus*	3	5	21.739
				*Moesziomyces*	4	2	8.696
				*Sakaguchia*	5	1	4.348
				**Total**		23	100
				**ANOVA**	0.002 **		

‘A’ represents the abundance of a given genera detected. ‘%’ is the percent that a genus represents of the total fungal or yeast community. ** abundance of genera among localities is highly significant (α = 0.001; *p* ≤ 0.001); *** abundance of genera among localities is extremely significant (α = 0).

## Data Availability

Sequence reads were deposited in the National Center for Biotechnology Information Sequence GenBank (NCBI GenBank; Accession Nos. MW633287 to MW633318 and MW670464 to MW670580). The R data that supports the conclusions of this article will be made available by the authors upon reasonable request.

## Supplementary Materials

The following are available online at https://www.mdpi.com/article/10.3390/microorganisms9061167/s1, Figure S1: Absolute abundances of microbes detected. Abundance of (A) filamentous fungi and (B) yeast per genus among the 8 sites in southwestern Trinidad, Table S1: Quantitative and qualitative characteristics of fungal isolates on 2% oil-amended media. Growth rate is given as diameter/mm/day. * represents an overgrown culture, and no measurement was taken. Visual observations key: eod = excellent oil degradation, lod = little/minor oil degradation, god = good oil degradation, pod = poor oil degradation, nod = no oil degradation, d = oil droplets, r = ring of clearance around fungi, b = oil blobs, p = oil pusher, w = waves in oil, and r of oil = ring of oil. Isolate being selected as a top performer for identification is indicated by Y = yes. Isolates not selected as top performers are indicated by N = no. Figure S2: Rank-abundance dominance (RAD) plots for genera distribution of (A) filamentous fungi and (B) yeast. The best model is represented by the bolded line. Null represents the MacArthur model; Preemption represents the geometric series of the Motomura model; Lognormal represents the lognormal distribution of Preston; Zipf represents the Zipf Model; Mandelbrot represents the Zipf-Mandelbrot Model, Table S2: Visual observations of bacterial co-culture isolates on 2% oil-amended media. R2A media supported bacterial growth, and PDA supported yeast growth. Visual characterization key: z = zones of clearance by bacteria, nz = no zone clearance by bacteria, sz = small zone of clearance by bacteria, bz = big zone of clearance by bacteria, NOB = no oil behind bacteria, and r of oil = ring of oil. Zones of clearance key: * = small zone of clearance, ** = medium zone of clearance, *** = massive zone of clearance, **** = entire plate cleared, negative sign = no zone of clearance but growth was greater in this media, and x = no zone of clearance and growth was similar, Figure S3: Diagnostics for NMDS. (A) and (C) Shepard plot for filamentous fungi and yeast, respectively, showing the success of the NMDS since individual blue points do not deviate from fitted nonparametric regression (red line); R^2^ values of 1 indicate that the points match the monotonically increasing line perfectly. (B) and (D) Goodness of fit plot for filamentous fungi and yeast, respectively, shows no genera contributing to stress, Table S3: Identification of fungi in the final dataset from 8 sites in southwestern Trinidad. GenBank accession information is provided, where two references are provided per species, Figure S4: ANOSIM analysis for (A) filamentous fungi and (B) yeast among the locations, Table S4: Identification of bacterial dataset from 8 sites in southwestern Trinidad. GenBank accession information is provided where two references are provided per species. Table S5: Identification of yeast dataset from 8 sites in southwestern Trinidad. GenBank accession information is provided, where two references are provided per species. Table S6: Hydrocarbon degrading microbes previously identified in Trinidad. Microbes in bold were identified in this study. Table S7: The Akaike information criterion (AIC) value for the genera rank abundance distribution models of the filamentous fungi and yeast communities. Table S8: Relative abundances of filamentous fungi and yeast in the 8 sites in southwestern Trinidad. Table S9: Diversity indices for filamentous fungal communities in the 8 sites in southwestern Trinidad. Table S10: Diversity indices for yeast communities in the 8 sites in southwestern Trinidad. Table S11: SIMPER analysis. Cumulative and overall contributions of key contributing genera responsible for the observed patterns between sites. For example, for the filamentous fungal community, differences between Pablito and Fyzabad are driven by *Aspergillus*, *Epicoccum*, *Roussoella*, *Eutypella*, *Sydowia*, *Trichoderma*, *Oudemansiella*, and *Paraconiothyrium* in descending order; together, these eight genera drive more than 70% of the difference between the communities in Pablito and Fyzabad; overall, the fungal communities in Pablito and Fyzabad are 91.7% different from each other.
