# Supplementary material for: Diversity and Oil Degradation Potential of Culturable Microbes Isolated from Chronically Contaminated Soils in Trinidad
Source: Microorganisms. 2021 May 28;9(6):1167. doi: 10.3390/microorganisms9061167 (PMC8230346; doi:10.3390/microorganisms9061167)
Supplement: Supplementary file 1 [file microorganisms-09-01167-s001.zip › microorganisms-1241727-SI.pdf]

Supplementary Material

1. Supplementary Data

1.1. Supplementary Figures

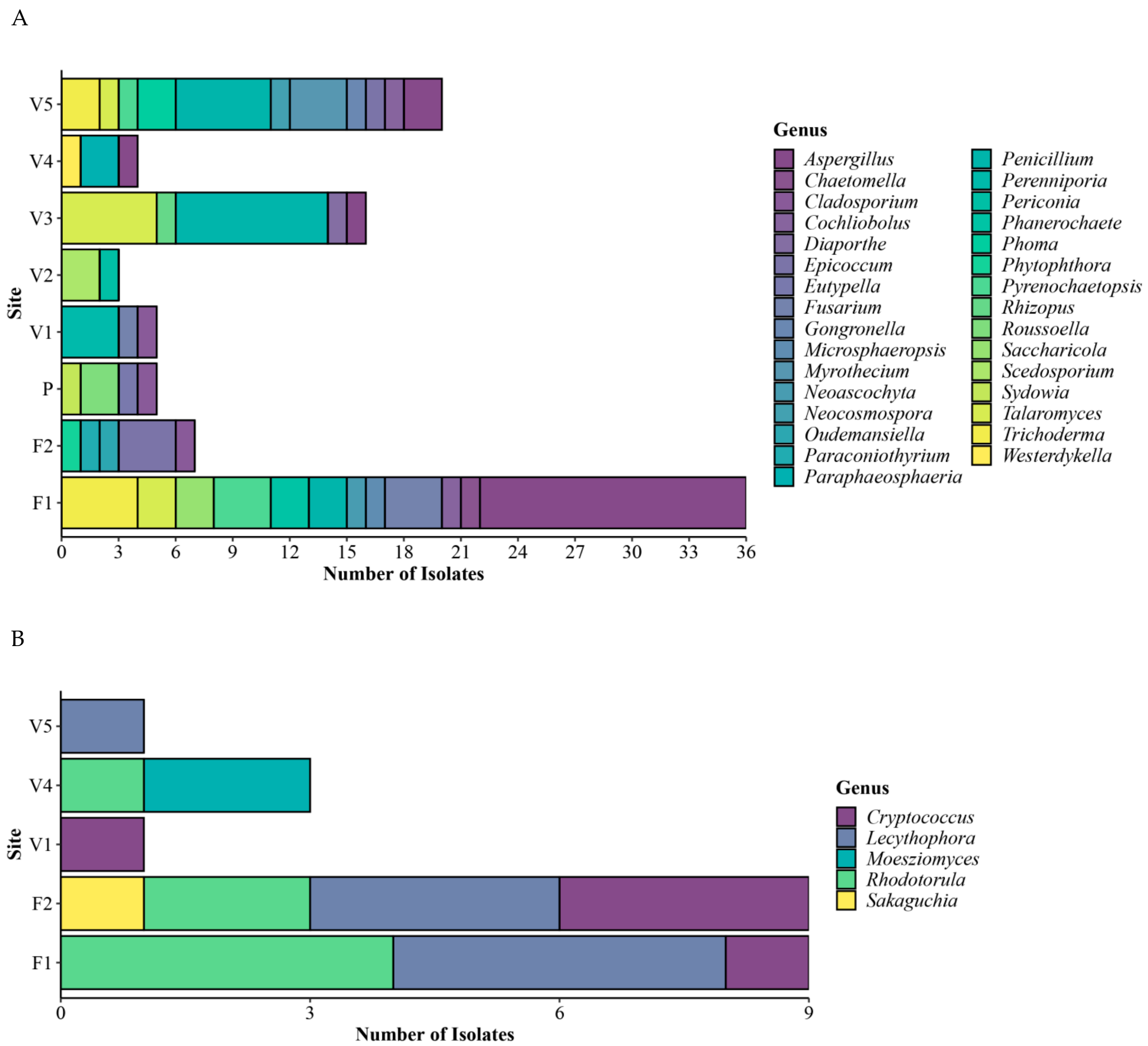

Figure S1: Absolute abundances of microbes detected. Abundance of: (A) filamentous fungi and (B) yeast per genus among the 8 sites in southwestern Trinidad.

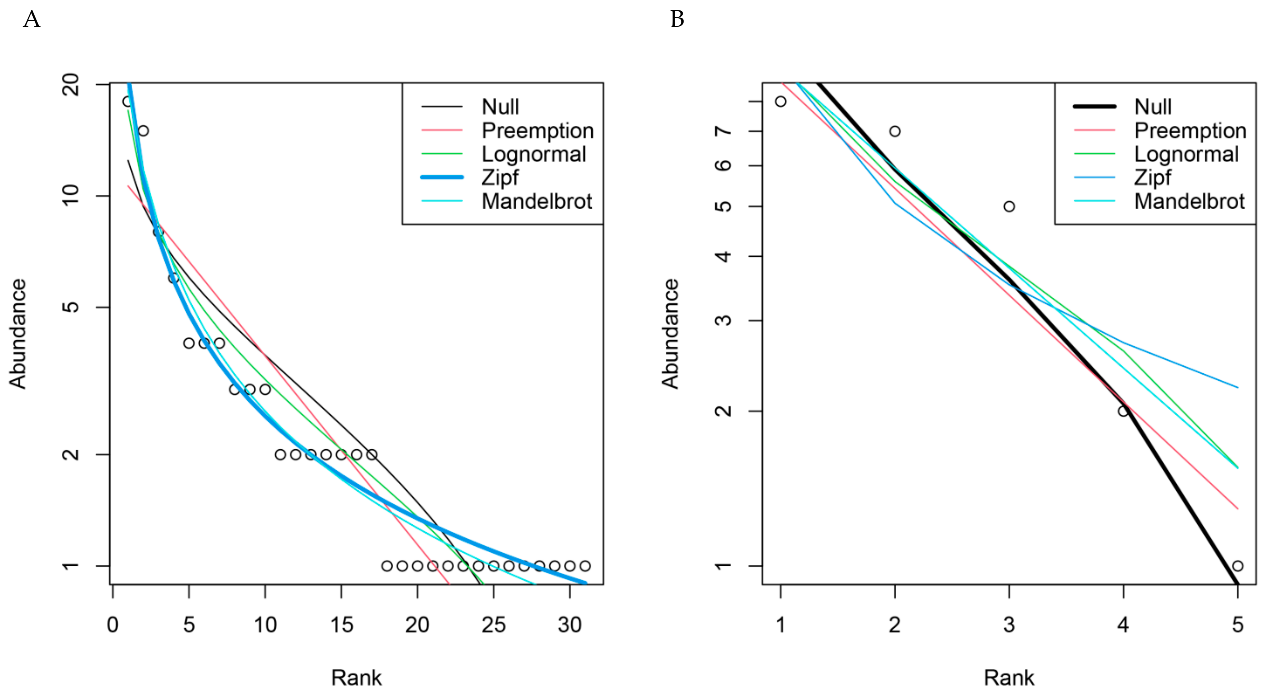

Figure S2: Rank-abundance dominance (RAD) plots for genera distribution of: (A) filamentous fungi and (B) yeast. The best model is represented by the bolded line. Null represents the MacArthur model; Preemption represents the geometric series of Motomura Model; Lognormal represents the Lognormal distribution of Preston; Zipf represents the Zipf Model; Mandelbrot represents the Zipf-Mandelbrot Model.

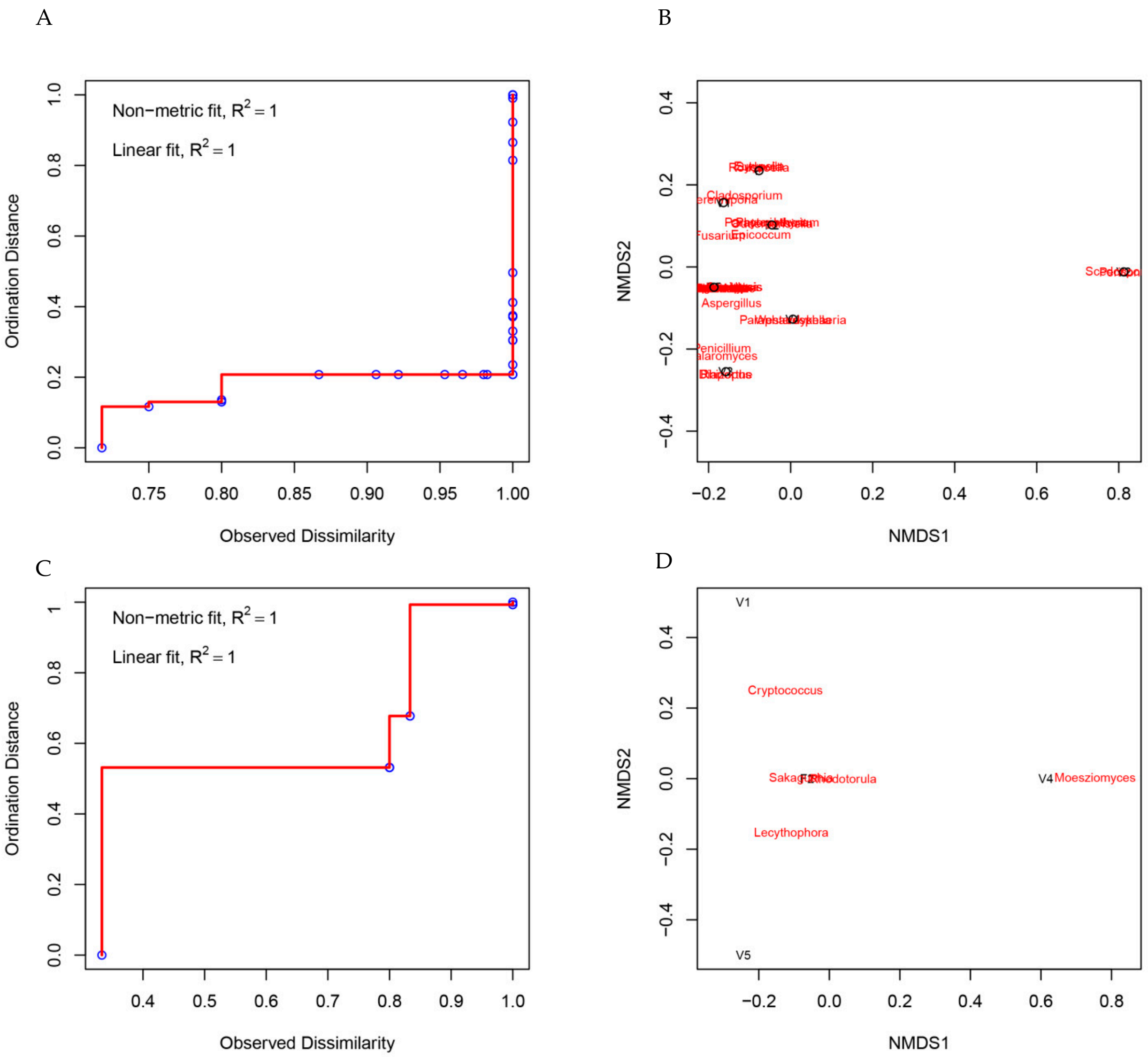

Figure S3: Diagnostics for NMDS. (A) and (C) Shepard plot for filamentous fungi and yeast respectively, showing success of the NMDS since individual blue points do not deviate from fitted non-parametric regression (red line); R^2 values of 1 indicate that the points match the monotonically increasing line perfectly. (B) and (D) Goodness of fit plot, for filamentous fungi and yeast respectively, shows no genera contributing to stress.

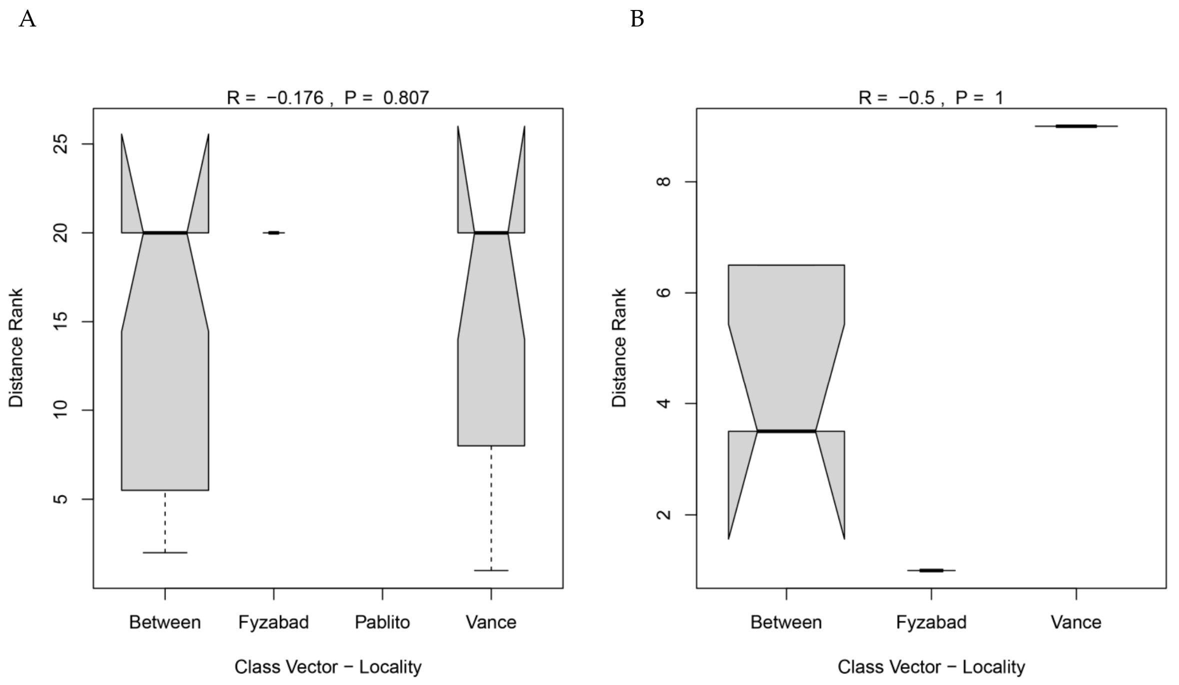

Figure S4: ANOSIM analysis for: (A) filamentous fungi and (B) yeast among the locations.

1.2. Supplementary Tables

Table S1: Quantitative and qualitative characteristics of fungal isolates on 2% oil-amended media. Growth rate is given as diameter/mm/day. * represents an overgrown culture and no measurement was taken. Visual observations key: eod = excellent oil degradation, lod = little/minor oil degradation, god good = oil degradation, pod = poor oil degradation, nod = no oil degradation, d = oil droplets, r = ring of clearance around fungi, b = oil blobs, p= oil pusher, w = waves in oil and r of oil = ring of oil. Isolate being selected as a top performer for identification is indicated by Y = Yes. Isolates not selected as top performers are indicated by N = no.

Site	Isolate No.	Growth rate on PDA	Observation	Growth rate on BHA	Observation	Keep
P	1	4.32	god	0.41	god	N
	2	10.63	pod	9.80	pod	Y
	3	2.36	god	2.91	god	Y
	4	1.84	eod	2.84	eod	Y
	5	2.13	r, eod	1.48	r, eod	N
	6	2.17	r, eod	2.14	r, eod	Y
F1	1	11.31	p, oil ring	7.78	d, lod	Y
	2	4.60	d, b, eod	2.22	d, r, god	Y
	3	*	lod, v tiny d	14.91	d, god	N
	4	10.02	b, god	7.59	d, lod	Y
	5	6.69	god	4.91	d, b	Y
	6	5.29	god	3.74	d, b, lod	Y
	7	3.83	god	1.96	d, r, lod	Y
	8	3.60	b,lod	1.46	d, r, god	N
	9	6.41	r, god	1.71	god	Y

10	6.65	d, eod	5.45	d, lod	N
11	6.90	r, d, eod	1.73	god	N
12	6.39	r, d, b, god	1.45	r, god	N
13	4.76	r, d, god	1.65	d, r, god	N
14	6.48	d, god	2.27	d, r, god	N
15	5.16	p, really god	5.22	b, lod	Y
16	6.66	god	5.53	d, god	Y
17	3.52	eod	2.22	d	N
18	6.27	god	5.62	d, b, lod	Y
19	3.05	eod	1.43	d, r, god	N
20	7.23	p, eod	6.75	d, lod	Y
21	3.31	b, r, god	1.36	d, r, god	N
22	2.06	r, god	1.90	d, b, lod	Y
23	5.14	r, eod	6.30	d, r, god	Y
24	3.98	really god	1.06	d, r, god	N
25	*	d, eod	14.91	d, r, god	Y
26	4.04	r, d, really god	3.50	d, r, god	Y
27	6.20	r, d, god	5.42	d, b	Y
28	1.34	NOB	0.85	d, god	Y
29	7.88	r, d, b, god	5.01	d, lod	Y
30	5.48	r, d, god	5.04	d, god, r of oil	Y
31	*	god	*	r, d, lod	Y
32	*		*	d, b, r, lod	Y
33	7.00	d, eod	7.95	d, b, r, lod	Y
34	*		*	r, lod	Y
35	3.35	r, d, god	3.76	d, b, r	Y
36	6.58	god	5.27	d, b, r	Y
37	6.43	p, eod	5.45	d, b, lod	Y
37A	9.05	god	11.77	d, b, lod	Y
38	3.86	r, eod	1.78	d, god	Y
39	2.19	p, r, god	1.61	d, lod	N
40	*	god	11.74	d, r, god	Y
41	2.66	r, b, lod	2.10	d, r, god	N
42	7.67	r, god	8.18	d, b, r, lod	Y
43	5.35	r, d, really god	5.14	b, god	Y
44	9.52	d, lod	8.46	d, b	Y
45	1.90	r, d, god	3.91	d, b, lod	Y
46	3.15	r, d, eod	4.42	p, d, b	Y

	47	11.88	b, lod	6.85	d, god	Y
	48	3.40	r, p, really god	3.57	d, god	Y
	49	1.88	lod	1.94	d, b, lod	Y
F2	1	5.46	r, d, god	3.83	d, god	Y
	1A	8.59	r, god	7.61	d,r, god	Y
	2	8.74	r, lod	8.55	b, d, r, lod	Y
	3	5.75	god	1.56	d, r, god	N
	4	8.39	r, god	7.61	b, d, r, god	Y
	5	1.63	r, really god	1.17	d, god	N
	5A	13.25	lod	13.72	d, b, lod	Y
	5B	1.80	r, eod	1.90	d, god	N
	6	6.23	really god	6.23	d, b, lod	Y
	7	1.91	r, eod	1.82	d, god	Y
	8	1.36	r, NOB	1.68	d, god	N
V1	1	5.23	lod	4.68	w, r	Y
	2	4.63	r, god	3.23	nod	Y
	3	10.29	god	11.80	d, nod	Y
	4	4.78	r, god	4.05	nod	Y
	5	3.36	god	2.95	god	Y
V2	1	3.68	r, god	4.29	d, b, lod	Y
	1*	-	-	4.41	d, lod	N
	1A	-	-	5.10	d, b, r, lod	N
	2	2.36	god	2.21	d, r, god	N
	3	3.95	god	2.11	d, r, god	N
	4	13.80	lod	3.94	d, r, god	Y
	5	3.52	god	4.72	d, r, god	Y
	6	5.91	god	1.93	d, r, god	N
	7	1.34	god	1.00	d, r, god	N
V3	1	6.09	god	3.95	d, w, nod	Y
	2	4.30	p, eod	3.70	d, w, nod	Y
	3	7.32	god	5.25	d, w, nod	Y
	4	6.75	god	5.52	d, nod	Y
	5	4.00	god	3.70	d, w, nod	Y
	6	5.39	god	1.91	eod	N
	7	7.00	god	2.50	d, god	N
	8	6.73	god	5.89	d, nod	Y
	9	4.55	w, r,god	1.39	z, nod	N
	10	6.09	god	4.30	p, d, nod	Y

	11	7.05	god	3.72	d, b, nod	Y
	12	7.18	lod	5.02	d, nod	Y
	13	4.77	god	1.68	nod	N
	14	6.76	god, r	6.02	d, nod	Y
	15	5.43	god, r	5.59	d, nod	Y
	16	2.20	god	2.66	d, nod	N
	17	4.41	god	3.97	d, b, r, nod	Y
	18	5.98	god, r	5.10	d, nod	Y
	19	12.03	god	5.53	d, nod	Y
V4	1	4.79	eod	6.89	d	Y
	2	3.20	eod	3.39	nod	N
	3	6.27	eod	7.11	nod	Y
	3*	-	-	5.11	d, nod	N
	4	5.32	eod	2.82	p	Y
	5	2.59	eod	2.48	r, eod	N
	6	4.10	eod	3.98	w	Y
	7	2.66	eod	2.86	r, eod	N
V5	1	6.26	really eod	4.14	d, nod	Y
	2	4.77	d, r, god	5.07	d, nod	Y
	3	7.77	god	8.16	v tiny d, nod	Y
	4	3.91	really eod	4.64	p, w	Y
	5	6.30	r, god	4.70	d,lod	Y
	6	8.18	god	9.91	w, d	Y
	7	4.25	eod	*	w, d	Y
	8	5.59	eod	4.36	p, lod	Y
	9	1.39	eod	5.20	d, nod	Y
	10	*	god	*	p, d, lod	Y
	11	4.87	god	6.00	d, r, nod	N
	11*			5.84	r, nod	Y
	12	4.98	god	7.91	r, nod	Y
	12*			8.00	d, lod	Y
	13	7.93	really god	5.82	r, nod	Y
	13A	7.89	lod	11.68	v tiny d, r	Y
	14	4.25	god	4.86	d, nod	Y
	15	*	god	*	d, p?	Y
	16	4.18	r,eod	4.41	d, nod	Y
	16*	-	-	4.39	w, god	Y
Total	128					

Table S2: Visual observations of bacterial co-culture isolates on 2% oil-amended media. R2A media supported bacterial growth and PDA supported yeast growth. Visual characterization key: z = zones of clearance by bacteria, nz = no zone clearance by bacteria, sz = small zone of clearance by bacteria, bz = big zone of clearance by bacteria, NOB = no oil behind bacteria and r of oil = ring of oil. Zones of clearance key: *= small zone of clearance, **= medium zone of clearance, ***= massive zone of clearance, ****= entire plate cleared, negative sign = no zone of clearance but growth was greater in this media and x = no zone of clearance and growth was similar.

		R2A				PDA		
		Characterization		Ranking		Characterization	Ranking	
Site	Isolate No.	Ab	w/o Ab	Ab	w/o Ab	Ab	Ab	Keep
P	1	z	nz, NOB, r of oil	*		z		Y
F1	1	NOB	NOB	-		nz, r		Y
	2	nz, NOB	NOB		-	nz, r		Y
	3	z	z, NOB		**	z	**	Y
	4	NOB	NOB	-		nz, NOB, r		Y
	5	nz, NOB	NOB	-		z	**	Y
	6	bz	bz		***	bz	****	Y
	7	nz, NOB	NOB, r of oil around		-	nz, r, NOB		Y
	8	NOB	NOB, r of oil around	x	x	nz, NOB, r		Y
	9	d, bz	nz, god	**		bz	****	Y
	10	very bz	bz	***		bz	****	Y
	11	bz	bz, lod	**		bz	****	Y
	12	bz	nz, lod	**		bz	****	Y
F2	1	NOB, r of oil	NOB, r of oil	-		nz, NOB, r		Y
	2	NOB, r of oil	NOB	-		nz, NOB, r		Y
	3	NOB, r of oil	NOB	-		nz, NOB, r		Y
	4	bz	z	**		bz	****	Y
	5	nz, NOB	NOB, r of oil	x	x	nz, NOB		Y
	6	bz, NOB	NOB, r of oil	**		nz		Y
	7	z	massive z, oil eaten and pushed		****	z	**	Y
	8	lod	bz, r of oillod		**	bz	****	Y
	9	NOB, r of oil	NOB, r of oil	x	x	nz, NOB, r		Y
	10	z	z	**		bz	****	Y
V1	1	z	massive z		****	z	**	Y
V2	1	z	massive z	***		z	**	Y
	2	z	massive z		****	z	**	Y
V3	1	z	massive z		****	nz		Y
	2	bz	r of oil, lod	**		bz	***	Y

V4	1	nz	nz, NOB, r of oil, sd	-		nz		Y
	2	bz	r of oil, NOB	**		z	**	Y
	3	NOB	r of oil, NOB	*		z	*	Y
V5	1	bz- 90% plate cleared	massive z, 95 % plate c no oil		****	z	**	Y
	2	z	massive z, 95 % plate c no oil		****	nz		Y
Total	33							

Table S3: Identification of filamentous fungi in the final dataset from 8 sites in southwestern Trinidad. GenBank accession information is provided where two references are provided per species. The placement of filamentous fungi into respective taxonomic levels was aided by the National Center for Biotechnology Information (NCBI) Taxonomy browser (https://www.ncbi.nlm.nih.gov/Taxonomy/Browser/wwwtax.cgi).

Site	Isolate No.	QC%/ID%	Species	Phyla	Class	Accession	Country	Host/source
P	2	99/99.82	*Eutypella scoparia*	Ascomycota	Sordariomycetes	KP269015	China	Seaweeds
		99/100				MK336539	Taiwan	Ipomoea pes-caprae
	3	100/99.43	*Roussoella solani*	Ascomycota	Dothideomycetes	LC195220	Japan	
						LC195219	Japan	
	4	99/99.46	*Roussoella solani*	Ascomycota	Dothideomycetes			
	6	100/99.62	*Cladosporium cladosporioides/pseudocladosporioides /subuliforme/allicinum/delicatulum/australiense/ angustisporum /inversicolor/westerdijkieae/uwebrauniana/europaeum/anthropophilum*	Ascomycota	Dothideomycetes			
F1	1	100/99.62	*Fusarium chlamydosporum*	Ascomycota	Dothideomycetes			
	2	100/100	*Aspergillus alabamensis*	Ascomycota	Sordariomycetes	KP987071	USA	Human/wound
						MG461687	China	Ceramium japonicum
	4	100/100	*Aspergillus caelatus*	Ascomycota	Eurotiomycetes	KM613140	Brazil	Soil
						AF004930	USA	Peanut field soil
	5	100/100	*Aspergillus aculeatus*	Ascomycota	Eurotiomycetes			
	6	100/100	*Aspergillus alabamensis*	Ascomycota	Eurotiomycetes			
	7	100/99.63	*Talaromyces angelicus*	Ascomycota	Eurotiomycetes	LT899791	Spain	Dung

					LT899792	Spain	Dung
9	100/100	*Penicillium janthinellum/brefeldianum*	Ascomycota	Eurotiomycetes			
15	100/100	*Aspergillus udagawae/wyomingensis/Neosartorya aureola*	Ascomycota	Eurotiomycetes	LC317463	Japan	
					KY808744	Australia	
16	100/100	*Aspergillus udagawae/wyomingensis/Neosartorya aureola*	Ascomycota	Eurotiomycetes			
18	100/100	*Aspergillus udagawae/wyomingensis/Neosartorya aureola*	Ascomycota	Eurotiomycetes			
20	100/99.62	*Fusarium chlamydosporum*	Ascomycota	Eurotiomycetes	KX421422	Brazil	Chrysomelidae sp.
					MN882831	Nigeria	Dried food
22	98/100	*Penicillium rubidurum*	Ascomycota	Eurotiomycetes	KP055596	South Korea	Crop field soil
					HQ607978	USA	Cyphomyrmex wheeleri nest
23	100/100	*Microsphaeropsis arundinis*	Ascomycota	Dothideomycetes	MH911414	India	Loktak Lake
					MH911394	India	Loktak Lake
25	100/100	*Trichoderma reesei*	Ascomycota	Sordariomycetes			
26	100/97.02	*Pyrenochaetopsis indica*	Ascomycota	Dothideomycetes	LT623224	India	Saccharum officinarum

					NR_160058	Spain	
27	100/99.82	*Aspergillus udagawae/aureolus/wyomingensis*	Ascomycota	Eurotiomycetes			
28	100/100	*Aspergillus alabamensis*	Ascomycota	Eurotiomycetes			
29	100/100	*Aspergillus udagawae/wyomingensis/Neosartorya aureola/fischeri*	Ascomycota	Eurotiomycetes			
31	100/99.84	*Trichoderma reesei/Hypocrea jecorina*	Ascomycota	Sordariomycetes			
32	100/99.84	*Trichoderma reesei/Hypocrea jecorina*	Ascomycota	Sordariomycetes			
33	100/100	*Aspergillus aculeatus/japonicus/fijiensis/brunneoviolaceus/assiutensis*	Ascomycota	Eurotiomycetes	KR296861	Singapore	Soil
					MK392046	China	
34	100/100	*Trichoderma reesei/Hypocrea jecorina*	Ascomycota	Eurotiomycetes	JQ411369	Panama	Marine sponge
					KM246746	Malaysia	
35	100/97	*Pyrenochaetopsis indica*	Ascomycota	Dothideomycetes			
36	100/100	*Talaromyces flavus var. flavus/muroii*	Ascomycota	Eurotiomycetes			
37	100/100	*Aspergillus udagawae/wyomingensis/Neosartorya aureola/fischeri*	Ascomycota	Eurotiomycetes			
37A	100/100	*Fusarium proliferatum/verticillioides/oxysporum/fujikuroi/mexicanum /napiforme/pseudocircinatum/Curvularia senegalensis*	Ascomycota	Eurotiomycetes			
38	100/99.83	*Aspergillus alabamensis*	Ascomycota	Eurotiomycetes			
40	100/99.83	*Phanerochaete chrysosporium/concrescens*	Basidiomycota	Agaricomycetes	KC881189	Argentina	Platanus acerifolia/wood
					GQ280374	Mexico	Wood wastes

	42	100/100	*Cochliobolus geniculatus/asianensis*	Ascomycota	Dothideomycetes	JQ783058	Brazil	Seedling sugarcane
						JN943417	Japan	Leaves
	43	99/99.77	*Chaetomella raphigera*	Ascomycota	Leotiomycetes	KF193633	China	Leaves
						AY487076	USA	
	44	99/98.05	*Stagonospora bicolor*	Ascomycota	Dothideomycetes			
	46	99/97.17	*Pyrenochaetopsis indica*	Ascomycota	Dothideomycetes			
	47	100/98.06	*Stagonospora bicolor/Saccharicola bicolor*	Ascomycota	Eurotiomycetes	MK102692	China	Stipa purpurea/root
						MK102691	China	Stipa purpurea/root
	48	100/100	*Aspergillus terreus*	Ascomycota	Eurotiomycetes	KP987086	China	Waste cloth
						JF738047	China	
	49	100/95.70	*Neoascochyta paspali*	Ascomycota	Dothideomycetes	MH861378	New Zealand	
						MN077412	New Zealand	Ranunculus acris/ leaf, symptomless tissue
F 2	1	81/93.61	*Paraconiothyrium variabile*	Ascomycota	Dothideomycetes	KR909137	USA	Grapevine/wood
						JX456476	Greece	Phoenix theophrasti/leaves

	1A	100/100	*Epicoccum sorghinum/latusicollum/thailandicum/Cochliobolus kusanoi= Curvularia kusanoi/Phoma herbarum/Fusarium oxysporum*	Ascomycota	Dothideomycetes	MF580966	China	Macadamia
						KX171660	Denmark	Sorghum bicolor/root
	2	100/100	*Epicoccum sorghinum/Cochliobolus kusanoi=Curvularia kusanoi/Phoma herbarum*	Ascomycota	Dothideomycetes			
	4	100/100	*Epicoccum sorghinum/latusicollum/thailandicum Cochliobolus kusanoi= Curvularia kusanoi/Phoma herbarum/Fusarium oxysporum*	Ascomycota	Dothideomycetes			
	6	99/99.84	*Phlebiopsis flavidoalba/ Oudemansiella canarii*	Basidiomycota	Agaricomycetes	KP135404	USA	Hardwood
						MG751231	Brazil	Hevea brasiliensis
	7	100/100	*Cladosporium sphaerospermum/Cladosporium halotolerans*	Ascomycota	Dothideomycetes	KX958086	Japan	Sediment below ocean floor
						KP269061	China	Seaweeds
V1	1	100/93.06	*Perenniporia medulla-panis*	Basidiomycota	Agaricomycetes			
	2	100/93.07	*Perenniporia medulla-panis*	Basidiomycota	Agaricomycetes			
	3	100/93.08	*Perenniporia medulla-panis*	Basidiomycota	Agaricomycetes	FJ627262	China	
						KX778655	China	Fishscale bamboo
	4	100/100	*Fusarium proliferatum(=Gibberella intermedia)*	Ascomycota	Eurotiomycetes	KM369868	India	Mangrove rhizosphere soil
						KJ207393	Hong Kong	Sticks in soil
	5	100/100	*Cladosporium cladosporioides*	Ascomycota	Dothideomycetes	HQ327999	China	

						KU707926	India	Metal rich rhizospheric soil
V2	1	100/99.83	*Scedosporium dehoogii/Pseudallescheria boydii*	Ascomycota	Sordariomycetes			
	4	100/98.51	*Periconia thailandica*	Ascomycota	Dothideomycetes	KY753887	Thailand	Decaying bamboo
		95/99				MN398986	Vietnam	
	5	100/99.83	*Scedosporium dehoogii*	Ascomycota	Sordariomycetes	KP132700	France	
						KX664394	USA	Floor surface
V3	1	100/99.62	*Talaromyces flavus/flavus var. flavus*	Ascomycota	Eurotiomycetes	JN602366	China	
						JX677940	Myanmar	Paddy soil
	2	100/99.82	*Penicillium shearii*	Ascomycota	Eurotiomycetes			
	3	100/99.82	*Talaromyces flavus/Talaromyces pinophilus (formerly Penicillium pinophilum)/liani*	Ascomycota	Eurotiomycetes	AF033420	USA	
						MH858867	Cote d'Ivoire	
	4	100/100	*Talaromyces flavus var. flavus*	Ascomycota	Eurotiomycetes			
	5	100/100	*Penicillium shearii*	Ascomycota	Eurotiomycetes			
	8	100/98.93	*Penicillium janthinellum/levitum/reticulisporum/ludwigii/ehrlichii*	Ascomycota	Eurotiomycetes			
	10	100/99.82	*Penicillium citrioviride*	Ascomycota	Eurotiomycetes			
	11	100/100	*Penicillium janthinellum/javanicum*	Ascomycota	Eurotiomycetes			
	12	100/100	*Talaromyces flavus var. flavus/muroii*	Ascomycota	Eurotiomycetes	MH857785	Netherlands	
						MH857477	Netherlands	

	14	100/99.47	*Penicillium citrioviride*	Ascomycota	Eurotiomycetes	GU388431	China	
						KM458837	India	Rhizospheric soil
	15	100/98.76	*Penicillium janthinellum/lineolatum/paneum/javanicum/levitum/reticulisporum*	Ascomycota	Eurotiomycetes			
	17	67/98.90	*Talaromyces macrosporus/aurantiacus*	Ascomycota	Eurotiomycetes	KU204425	Costa Rica	Oxalis frutescens (Oxalidaceae)
						MF039205	Brazil	Cocoa fermentation
	18	100/99.82	*Penicillium citrioviride*	Ascomycota	Eurotiomycetes			
	19	100/99.81	*Diaporthe phaseolorum*	Ascomycota	Sordariomycetes	JN541222	Ecuador	Coussapoa trinervia
						HM855215	Ecuador	Pourouma tomentosa
V4	1	99/93.15	*Paraphaeosphaeria arecacearum*	Ascomycota	Dothideomycetes			
	3	99/99.82	*Paraphaeosphaeria arecacearum*	Ascomycota	Dothideomycetes	JX496100	Netherlands	Cocos nucifera
		99/99.82				KM873041	India	Metal rich rhizospheric soil
	4	100/98.76	*Westerdykella dispersa/Westerdykella multispora*	Ascomycota	Dothideomycetes	KU702711	Lithuania	Sludge/digestate
						NR_111187	Virginia	Phlox drummondii seedling
	6	100/100	*Aspergillus stromatoides/Chaetosartorya stromatoides*	Ascomycota	Eurotiomycetes	NR_137454	USA	

						EF652146	USA	*Aspergillus stromatoides*
V5	1	100/100	*Penicillium javanicum/janthinellum*	Ascomycota	Eurotiomycetes			
	2	100/100	*Penicillium javanicum/janthinellum*	Ascomycota	Eurotiomycetes	MH864718	Brazil	
						KF313084	Korea	Pine root
	3	100/100	*Aspergillus nomius*	Ascomycota	Eurotiomycetes	AB828718	Thailand	Soil contaminated palm oil
						MH279387	Thailand	Bamboo sample
	4	100/100	*Penicillium citrinum*	Ascomycota	Eurotiomycetes	MG948252	India	Soil
						KT844552	India	Rhizospheric soil from agricultural fields
	5	100/100	*Gongronella butleri*	Mucoromycota	Mucoromycetes	JN942999	Brazil	Soil
						KM405649	India	Medicinal plant
	6	100/99.80	*Epicoccum sorghinum (syn Epicoccum sorghi)/Phoma herbarum /Epicoccum latusicollum*	Ascomycota	Dothideomycetes			
	7	99/97.74	*Penicillium pulvillorum*	Ascomycota	Eurotiomycetes	AF178527	USA	
						KF624805	USA	Ant mound
	8	99/100	*Penicillium javanicum/janthinellum*	Ascomycota	Eurotiomycetes			
	9	100/100	*Pyrenochaetopsis microspora/americana/Dokmaia monthadangii /Melanomma lichenicola*	Ascomycota	Dothideomycetes	MK508814	Brazil	Leaves of coconut crops

					NR_160059	USA	
10	100/100	*Trichoderma asperellum/asperelloides/yunnanense /hamatum*	Ascomycota	Sordariomycetes	MK086064	Brazil	Soil
					MN639282	Mexico	Soil
11	100/99.41	*Curvularia kusanoi*	Ascomycota	Dothideomycetes	JN943395	Japan	Eragrostis multicaulis/leaves
					MG847354	China	Oedaleus infernalis Sauss gut
12	100/100	*Phoma herbarum/Epicoccum sorghinum/Cochliobolus kusanoi*	Ascomycota	Dothideomycetes			
12*	100/100	*Phoma herbarum/Epicoccum sorghinum/Cochliobolus kusanoi*	Ascomycota	Dothideomycetes	KY780194	China	Leaf
					KU204761	South Africa	Endophytes
13	100/100	*Talaromyces pinophilus/funiculosus/cellulolyticus*	Ascomycota	Eurotiomycetes	LC199384	Denmark	Deep sea sediment
					KY965441	China	Roots of Dalbergia odorifera
13A	100/100	*Neocosmospora rubicola*	Ascomycota	Sordariomycetes	KY283805	China	Orchid
14	100/100	*Myrothecium gramineum*	Ascomycota	Sordariomycetes	FJ825374	Thailand	
					JQ936266	Brazil	Soybean/leaves
15	100/99.83	*Trichoderma reesei=Hypocrea jecorina*	Ascomycota	Sordariomycetes			
16	99/99.64	*Myrothecium roridum*	Ascomycota	Sordariomycetes	JF724155	Brazil	Leaf

						AJ302001	Germany	
	16*	100/99.82	*Myrothecium roridum*	Ascomycota	Sordariomycetes			
Isolates from co-culture								
P	1	99/98.23	*Sydowia sp.*	Ascomycota	Dothideomycetes	MF683457	New Zealand	Kunzea ericoides
F1	2	99/76.90	*Phanerochaete chrysosporium*	Basidiomycota	Agaricomycetes			
F2	7	6/95.12	*Phytophthora cinnamomi*	Oomycota	Oomycetes	EU170011	Australia	
V3	1	92/100	*Aspergillus gracilis*	Ascomycota	Eurotiomycetes	MF920422	Antarctica	Human impacted soil
						MF692973	Antarctica	Pristine soil
	2	75/77.88	*Rhizopus oryzae*	Mucoromycota	Mucoromycetes	EU862193	China	Traditional soy paste and soy sauce
V5	1	73/82.48	*Aspergillus sydowii*	Ascomycota	Eurotiomycetes	MH464419	China	During fermentation
						MK828710	Nigeria	Untreated refinery wastewater
**Total**	**96**							

Table S4: Identification of bacterial dataset from 8 sites in southwestern Trinidad. GenBank accession information is provided where two references are provided per species. The placement of bacteria into respective taxonomic levels was aided by the National Center for Biotechnology Information (NCBI) Taxonomy browser (https://www.ncbi.nlm.nih.gov/Taxonomy/Browser/wwwtax.cgi).

Site	Isolate No.	Shape	Gram reaction	QC%/ID%	Identity	Phyla	Class	Accession	Country	Host/source
P	1	Bacilli	-	100/99.46	*Janthinobacterium lividum/svalbardensis*	Proteobacteria	Betaproteobacteria			
F1	1	Bacilli	-	100/99.78	*Burkholderia anthina/cepacia*	Proteobacteria	Betaproteobacteria	KP216607	China	Rhizosphere soil of the different ages Dalbergia Odorifera
								JX025731	India	Soil
	2	Bacilli	-	100/99	*Burkholderia cenocepacia/cepacia*	Proteobacteria	Betaproteobacteria	KY810685	India	Root
								EU684748	China	
	3	Bacilli	-	89/82.45	*Burkholderia gladioli*	Proteobacteria	Betaproteobacteria	KJ670084	Brazil	Sugarcane endophytic
								MG654707	China	
	4	Bacilli	-	100/98.33	*Janthinobacterium lividum/svalbardensis*	Proteobacteria	Betaproteobacteria			
	5	Bacilli	-	100/99.90	*Burkholderia cepacia/territorii/ambifaria/cenocepacia/pyrrocinia/metallica/vietnamiensis*	Proteobacteria	Betaproteobacteria	MG768915	Vietnam	Dioxin contaminated soils
								MG768914	Vietnam	Dioxin contaminated soils

	6	Bacilli	-	100/99.47	*Chryseobacterium oranimense*	Bacteroidetes	Flavobacteriia	NR_044168	Israel	Raw milk
								MG322209	Portugal	Surface water
	7	Bacilli	-	100/99.67	*Janthinobacterium lividum/svalbardensis*	Proteobacteria	Betaproteobacteria			
	8	Bacilli	-	100/100	*Janthinobacterium lividum*	Proteobacteria	Betaproteobacteria	MG825076	Antarctica	Hydrocarbon-contaminated soil
								KT923309	Austria	Alpine soil-Hydrocarbon-degrading bacteria
	9	Bacilli	-	99/84.49	*Janthinobacterium lividum*	Proteobacteria	Betaproteobacteria			
	10	Bacilli	-	100/100	*Janthinobacterium lividum/svalbardensis*	Proteobacteria	Betaproteobacteria			
	11	Bacilli	-	99/99.69	*Janthinobacterium lividum*	Proteobacteria	Betaproteobacteria			
	12	Bacilli	-	100/99.68	*Janthinobacterium lividum/svalbardensis*	Proteobacteria	Betaproteobacteria			
F2	1	Bacilli	-	100/97.36	*Janthinobacterium lividum/svalbardensis*	Proteobacteria	Betaproteobacteria			
	2	Bacilli	-	100/99.15	*Janthinobacterium lividum*	Proteobacteria	Betaproteobacteria			
	3	Bacilli	-	99/99.18	*Janthinobacterium lividum*	Proteobacteria	Betaproteobacteria			
	4	Bacilli	-	100/99.89	*Janthinobacterium lividum/svalbardensis*	Proteobacteria	Betaproteobacteria			
	5	Bacilli	-	100/100	*Janthinobacterium lividum*	Proteobacteria	Betaproteobacteria			
	6	Bacilli	-	100/99.76	*Janthinobacterium lividum/svalbardensis*	Proteobacteria	Betaproteobacteria			
	7	Bacilli	-	100/99.69	*Serratia marcescens/nematodiphila*	Proteobacteria	Gammaproteobacteria	KP903465	China	Rhizosphere soil of Camellia sinensis
								KM044037	Inida	Soil

	8	Bacilli	-	100/99.88	*Janthinobacterium lividum/svalbardensis*	Proteobacteria	Betaproteobacteria			
	9	Bacilli	-	99/100	*Janthinobacterium lividum/svalbardensis*	Proteobacteria	Betaproteobacteria			
	10	Bacilli	-	100/100	*Janthinobacterium lividum/svalbardensis*	Proteobacteria	Betaproteobacteria			
V1	1	Bacilli	-	100/97.12	*Janthinobacterium lividum/svalbardensis*	Proteobacteria	Betaproteobacteria			
V2	1	Bacilli	-	100/99.78	*Serratia marcescens/entomophila*	Proteobacteria	Gammaproteobacteria			
	2	Bacilli	-	100/99.49	*Serratia marcescens*	Proteobacteria	Gammaproteobacteria			
V3	1	Bacilli	-	100/99.68	*Serratia nematodiphila*	Proteobacteria	Gammaproteobacteria			
	2	Bacilli	-	99/100	*Serratia nematodiphila*	Proteobacteria	Gammaproteobacteria	GU339284	Inida	
								MN691570	China	Rice paddy soil
V4	1	Bacilli	-	99/88.28	*Janthinobacterium lividum*	Proteobacteria	Betaproteobacteria			
	2	Bacilli	-	99/95.11	*Janthinobacterium lividum*	Proteobacteria	Betaproteobacteria			
	3	Bacilli	-	100/100	*Janthinobacterium lividum/svalbardensis*	Proteobacteria	Betaproteobacteria			
V5	1	Bacilli	-	100/99.89	*Serratia marcescens*	Proteobacteria	Gammaproteobacteria			
	2	Bacilli	-	100/99.79	*Janthinobacterium lividum*	Proteobacteria	Betaproteobacteria			
**Total**	**33**									

Table S5: Identification of yeast dataset from 8 sites in southwestern Trinidad. GenBank accession information is provided where two references are provided per species. The placement of yeast into respective taxonomic levels was aided by the National Center for Biotechnology Information (NCBI) Taxonomy browser (https://www.ncbi.nlm.nih.gov/Taxonomy/Browser/wwwtax.cgi).

Site	Isolate No.	QC%/ID%	Identity	Phyla	Class	Accession	Country	Host/source
F1	3	81/96.41	*Cryptococcus rajasthanensis*	Basidiomycota	Agaricomycetes	AM262325	India	Infloresence of Digera sp.
						HQ832836	China	Camellia sinensis/ Foliar lesions
	4	100/100	*Rhodotorula mucilaginosa*	Basidiomycota	Microbotryomycetes	KU145520	Chile	Soil sample from Snow island
						KP132584	France	
	6	95/88.75	*Rhodotorula mucilaginosa*	Basidiomycota	Microbotryomycetes			
	7	100/100	*Rhodotorula mucilaginosa*	Basidiomycota	Microbotryomycetes			
	8	100/100	*Rhodotorula mucilaginosa*	Basidiomycota	Microbotryomycetes			
	9	100/99.81	*Lecythophora aff. decumbens*	Ascomycota	Sordariomycetes	FN428890	Brazil	Sugar cane soil
						HQ631011		Saccharum officinarum
	10	100/99.82	*Lecythophora aff. decumbens*	Ascomycota	Sordariomycetes			
	11	100/99.81	*Lecythophora aff. decumbens*	Ascomycota	Sordariomycetes			
	12	100/99.64	*Lecythophora aff. decumbens*	Ascomycota	Sordariomycetes			
F2	1	100/100	*Rhodotorula mucilaginosa*	Basidiomycota	Microbotryomycetes			
	2	94/99.80	*Cryptococcus flavescens*	Basidiomycota	Tremellomycetes	FN428902	Brazil	Sugar cane soil
						FN428920	Brazil	Sugar cane soil

	3	100/99.18	*Cryptococcus/Papiliotrema aff. laurentii*	Basidiomycota	Tremellomycetes			
	4	100/99.44	*Lecythophora aff. decumbens*	Ascomycota	Sordariomycetes			
	5	100/100	*Cryptococcus laurentii*	Basidiomycota	Tremellomycetes	KT899784	Nigeria	Crude oil polluted soil
						JN627015	Brazil	
	6	100/100	*Rhodotorula taiwanensis/glutinis*	Basidiomycota	Microbotryomycetes	LC486521	Thailand	Rice leaves (Oryza sativa)
						LC191386	Thailand	Corn leaves
	8	100/99.63	*Lecythophora aff. decumbens*	Ascomycota	Sordariomycetes			
	9	100/95.30	*Sakaguchia dacryoidea*	Basidiomycota	Cystobasidiomycetes	AF444571		
						AF444500		
	10	100/99.63	*Lecythophora aff. decumbens*	Ascomycota	Sordariomycetes			
V1	1	80/100	*Cryptococcus flavescens*	Basidiomycota	Tremellomycetes			
V4	1	99/100	*Pseudozyma sp.*	Basidiomycota	Ustilaginomycetes			
	2	99/96.32	*Moesziomyces parantarcticus=Pseudozyma parantarctica*	Basidiomycota	Ustilaginomycetes	KP132543	France	
						KF619567	Nigeria	Yam tuber steep water
	3	84/83.59	*Rhodotorula toruloides=Rhodosporidium toruloides*	Basidiomycota	Microbotryomycetes	FJ515214	Taiwan	Sea surface microlayer and underlying water
						MG050093	India	Soil
V5	2	100/99.81	*Lecythophora aff. decumbens*	Ascomycota	Sordariomycetes			
**Total**	**23**							

Table S6: Hydrocarbon degrading microbes previously identified in Trinidad. Microbes in bold were identified in this study.

Microbes	Locality	Citation
**Fungi**		
Main fungi: ***Aspergillus***, *Curvularia*, ***Fusarium***, ***Penicillium*** and *Mucor*	La Brea Pitch Lake; Digity Seepage	[85]
**Bacteria**		
Main bacteria: *Bacillus* spp., *Achromobacter* spp., ***Burkholderia*** spp., *Pseudomonas* spp., *Acidobacteria*	La Brea Pitch Lake; Digity Seepage	[85]
Burkholderiales, Enterobacteriales, Bacteriodales, Rhodospirillales, Sphingomonadales, Thermotogales, Nitrosomonadales, Actinomycetales, Hydrogenophilales, Neisseriales, Clostridiales, Pseudomonadales, Anaerolineales, Methanosarcinales, Thermoplasmatales, Methanomicrobiales, Halobacteriales	La Brea Pitch Lake	[83]
Thiotricales and *Campylo-bacteriales, Defferibacterales, Thermodesulfobacteriales, Nitrospirales, Desulfurimonadales,* and *Desulfobacterales, Pseudomonadales, Oceanospirillales, Burkholderiales, Acidobacteriales* and *Rhodospirillales*	La Brea Pitch Lake	[84]
Burkholderia sp., *Achromobacter* sp., *Leifsonia* sp., *Dyemonas* sp., *Pseudomans* sp., *Paenibacillus* sp.	Soil neighbouring La Brea Pitch Lake	[82]
**Archaea**		
Thermoplasmatales		[84]

Table S7: The Akaike information criterion (AIC) value for the genera rank abundance distribution models of the filamentous fungi and yeast communities.

Microbe	No. of genera	Abundance	Null	Preemption	Lognormal	Zipf	Mandelbrot
Fungi	31	96	98.581	100.02	93.557	88.831	90.219
Yeast	5	23	17.175	19.097	21.106	22.340	22.858

Table S8: Relative abundances of filamentous fungi and yeast in the 8 sites in southwestern Trinidad.

Genera	P	F1	F2	V1	V2	V3	V4	V5
**Fungi**								
Aspergillus	0.00	38.89	0.00	0.00	0.00	6.25	25.00	10.00
Chaetomella	0.00	2.78	0.00	0.00	0.00	0.00	0.00	0.00
Cladosporium	20.00	0.00	14.29	20.00	0.00	0.00	0.00	0.00
Cochliobolus	0.00	2.78	0.00	0.00	0.00	0.00	0.00	5.00
Diaporthe	0.00	0.00	0.00	0.00	0.00	6.25	0.00	0.00
Epicoccum	0.00	0.00	42.86	0.00	0.00	0.00	0.00	5.00
Eutypella	20.00	0.00	0.00	0.00	0.00	0.00	0.00	0.00
Fusarium	0.00	8.33	0.00	20.00	0.00	0.00	0.00	0.00
Gongronella	0.00	0.00	0.00	0.00	0.00	0.00	0.00	5.00
Microsphaeropsis	0.00	2.78	0.00	0.00	0.00	0.00	0.00	0.00
Myrothecium	0.00	0.00	0.00	0.00	0.00	0.00	0.00	15.00
Neoascochyta	0.00	2.78	0.00	0.00	0.00	0.00	0.00	0.00
Neocosmospora	0.00	0.00	0.00	0.00	0.00	0.00	0.00	5.00
Oudemansiella	0.00	0.00	14.29	0.00	0.00	0.00	0.00	0.00
Paraconiothyrium	0.00	0.00	14.29	0.00	0.00	0.00	0.00	0.00
Paraphaeosphaeria	0.00	0.00	0.00	0.00	0.00	0.00	50.00	0.00
Penicillium	0.00	5.56	0.00	0.00	0.00	50.00	0.00	25.00
Perenniporia	0.00	0.00	0.00	60.00	0.00	0.00	0.00	0.00
Periconia	0.00	0.00	0.00	0.00	33.33	0.00	0.00	0.00
Phanerochaete	0.00	5.56	0.00	0.00	0.00	0.00	0.00	0.00
Phoma	0.00	0.00	0.00	0.00	0.00	0.00	0.00	10.00
Phytophthora	0.00	0.00	14.29	0.00	0.00	0.00	0.00	0.00
Pyrenochaetopsis	0.00	8.33	0.00	0.00	0.00	0.00	0.00	5.00
Rhizopus	0.00	0.00	0.00	0.00	0.00	6.25	0.00	0.00
Roussoella	40.00	0.00	0.00	0.00	0.00	0.00	0.00	0.00
Saccharicola	0.00	5.56	0.00	0.00	0.00	0.00	0.00	0.00
Scedosporium	0.00	0.00	0.00	0.00	66.67	0.00	0.00	0.00
Sydowia	20.00	0.00	0.00	0.00	0.00	0.00	0.00	0.00
Talaromyces	0.00	5.56	0.00	0.00	0.00	31.25	0.00	5.00
Trichoderma	0.00	11.11	0.00	0.00	0.00	0.00	0.00	10.00
Westerdykella	0.00	0.00	0.00	0.00	0.00	0.00	25.00	0.00
**Yeast**								
Cryptococcus	0.00	11.11	33.33	100.00	0.00	0.00	0.00	0.00
Lecythophora	0.00	44.44	33.33	0.00	0.00	0.00	0.00	100.00
Moesziomyces	0.00	0.00	0.00	0.00	0.00	0.00	66.67	0.00
Rhodotorula	0.00	44.44	22.22	0.00	0.00	0.00	33.33	0.00
Sakaguchia	0.00	0.00	11.11	0.00	0.00	0.00	0.00	0.00

Table S9: Diversity indices for filamentous fungal communities in the 8 sites in southwestern Trinidad.

		Diversity		Richness		Dominance		Evenness
**Site**	**N**	**H**	**D_1**	***S***	**Chao 1**	**Absolute**	**D_2**	**$E_{1/D}$**
P	5	1.332	0.720	4	8.500	2	0.280	0.893
F1	36	2.066	0.807	12	14.000	14	0.193	0.432
F2	7	1.475	0.735	5	11.000	3	0.265	0.754
V1	5	0.950	0.560	3	4.000	3	0.440	0.758
V2	3	0.637	0.444	2	2.500	2	0.556	0.900
V3	16	1.230	0.641	5	8.000	8	0.359	0.557
V4	4	1.040	0.625	3	5.000	2	0.375	0.889
V5	20	2.221	0.870	11	17.000	5	0.130	0.699
**ANOVA**	0.858	<2e-16 ***	<2e-16 ***	0.002**	0.957	-	-	<2e-16 ***
**Locality**								
Pablito	5.000	1.332	0.720	4	8.500	2	0.280	0.893
Fyzabad	43.000	2.414	0.858	17	25.000	14	0.142	0.414
Vance	48.000	2.516	0.876	19	39.170	13	0.124	0.425
**ANOVA**	0.2655	-	-	0.255	0.24	-	-	-

Table S10. Diversity indices for yeast communities in the 8 sites in southwestern Trinidad.

		Diversity		Richness		Dominance		Evenness
**Site**	**N**	**H'**	**D_1**	***S***	**Chao 1**	**Absolute**	**D_2**	**$E_{1/D}$**
F1	9	0.965	0.593	3	3	4	0.407	0.818
F2	9	1.311	0.716	4	4.5	3	0.284	0.880
V1	1	0.000	0.000	1	1	1	1.000	1.000
V4	3	0.637	0.444	2	2.5	2	0.556	0.900
V5	1	0.000	0.000	1	1	1	1.000	1.000
**ANOVA**	0.069	<2e-16 ***	<2e-16 ***	0.164	0.242	-	-	<2e-16 ***
**Locality**								
Fyzabad	18	1.228	0.685	4	4	7	0.315	0.794
Vance	5	1.332	0.720	4	8.5	2	0.280	0.893
**ANOVA**	-	-	-	-	-	-	-	-

Table S11: SIMPER analysis. Cumulative and overall contributions of key contributing genera responsible for the observed patterns between sites. For example, for the filamentous fungal community, differences between Pablito and Fyzabad are driven by *Aspergillus, Epicoccum, Roussoella, Eutypella, Sydowia, Trichoderma, Oudemansiella* and *Paraconiothyrium* in descending order; together these eight genera drive more than 70% of the difference between communities in Pablito and Fyzabad; overall the filamentous fungal communities in Pablito and Fyzabad are 91.7% different from each other.

Microbial community	Locality	Genera	Cumulative contribution	Overall Contribution
**Fungi**	Pablito_Fyzabad	*Aspergillus*	0.186	0.917
		Epicoccum	0.323	
		Roussoella	0.440	
		Eutypella	0.499	
		Sydowia	0.558	
		Trichoderma	0.611	
		Oudemansiella	0.656	
		Paraconiothyrium	0.702	
	Pablito_Vance	*Roussoella*	0.177	0.960
		Penicillium	0.298	
		Eutypella	0.386	
		Sydowia	0.474	
		Cladosporium	0.542	
		Perenniporia	0.604	
		Talaromyces	0.662	
		Scedosporium	0.714	
	Fyzabad_Vance	*Apergillus*	0.186	0.915
		Epicoccum	0.300	
		Penicillium	0.393	
		Trichoderma	0.446	
		Talaromyces	0.499	
		Fusarium	0.542	
		Pyrenochaetopsis	0.581	
		Oudemansiella	0.620	
		Paraconiothyrium	0.658	
		Phytophthora	0.697	
		Perenniporia	0.733	
**Yeast**	Fyzabad_Vance	*Lecythophora*	0.366	0.811
		Rhodotorula	0.682	
		Cryptococcus	0.873	
